# The impact of unconditional cash transfers on morbidity and health-seeking behaviour in Africa: evidence from Ghana, Malawi, Zambia and Zimbabwe

**DOI:** 10.1093/heapol/czac014

**Published:** 2022-02-14

**Authors:** Jacob Novignon, Leah Prencipe, Adria Molotsky, Elsa Valli, Richard de Groot, Clement Adamba, Tia Palermo

**Affiliations:** Department of Economics, Kwame Nkrumah University of Science and Technology, Kumasi, Ghana; Department of Public Health, Erasmus Medical Center, Rotterdam 3000 CA, The Netherlands; American Institutes for Research, International Development Division, 1400 Crystal Drive, 10th Floor Arlington, VA 22202, USA; UNICEF Office of Research – Innocenti, Via degli Alfani 58, 50121 Florence, Italy; Independent Consultant, Josef Israelshof 23, Oosterhout 4907 PT, The Netherlands; Institute of Statistical, Social and Economic Research, University of Ghana-Legon, P.O. Box LG 74, Legon-Accra, Ghana; Department of Epidemiology and Environmental Health, State University of New York at Buffalo, 270 Farber Hall, Buffalo, NY 14214-800, USA

**Keywords:** Morbidity, health seeking, healthcare utilization, cash transfers, social protection, Africa

## Abstract

Unconditional cash transfers have demonstrated widespread, positive impacts on consumption, food security, productive activities and schooling. However, the evidence to date on cash transfers and health-seeking behaviours and morbidity is not only mixed, but the evidence base is biased towards conditional programmes from Latin America and is more limited in the context of Africa. Given contextual and programmatic design differences between the regions, more evidence from Africa is warranted. We investigate the impact of unconditional cash transfers on morbidity and health-seeking behaviour using data from experimental and quasi-experimental study designs of five government cash transfer programs in Ghana, Malawi, Zambia and Zimbabwe. Programme impacts were estimated using difference-in-differences models with longitudinal data. The results indicate positive programme impacts on health seeking when ill and on health expenditures. Our findings suggest that while unconditional cash transfers can improve health seeking when ill, morbidity impacts were mixed. More research is needed on longer-term impacts, mechanisms of impact and moderating factors. Additionally, taken together with existing evidence, our findings suggest that when summarizing the impacts of cash transfers on health, findings from conditional and unconditional programmes should be disaggregated.

**Key messages**

- Much of the evidence on cash transfers and health to date has come from Latin America, and evidence from unconditional cash transfers and Africa is more limited.
- More evidence from Africa is needed, given differences between this region and others with respect to higher levels of generalized poverty, higher infectious disease burden, lower service availability and differences in programme characteristics (e.g. conditional cash transfers in Latin America vs unconditional cash transfers in Africa).
- In this study using primary data from five government unconditional cash transfers in four African countries, we find that cash transfers have strong positive impacts on health seeking when ill and on health expenditures. However, we find limited impacts on morbidity, with significant variation by age group and country examined.

## Introduction

Social protection coverage has increased globally, with ∼46.9% of the global population covered by at least one benefit (ILO, 2021). In Africa, the number of unconditional cash transfer (UCT) programmes (one form of social protection) doubled to 40 between 2010 and 2015 (World Bank, 2015). Objectives of cash transfers (CTs) include alleviating high levels of poverty and food insecurity and to enable families to invest in human capital development (including health and education) to stem the persistence of poverty intergenerationally.

Given the high poverty levels and gaps in health and schooling infrastructure in many rural communities, government CT programmes in Africa tend to be unconditional, in contrast to conditional CT (CCT) programmes common in Latin America. CCT programmes generally require participants to comply with certain behavioural requirements, including health check-ups or school attendance, to maintain their eligibility. These differences in programme characteristics and contextual factors may have implications for programme impacts on population health outcomes.

In recent years, there has been a growing body of literature evaluating the impact of the various CT programmes in Africa (Bastagli *et al.*, 2019; Davis *et al.*, 2016; Hidrobo *et al.*, 2018; Owusu-Addo *et al.*, 2018; Onwuchekwa *et al.*, 2021). Findings from these studies suggest that the programmes achieve their immediate objectives of reducing poverty and improving food security among poor and vulnerable households (Bastagli *et al.*, 2019; Hidrobo *et al.*, 2018; Davis *et al.*, 2016). However, given the close linkages between poverty, food security and health, there could be potential gains related to health-seeking behaviours and outcomes. The focus of the current study is to evaluate the impact of UCT programmes on health seeking and health outcomes across four countries in Africa.

The study was motivated by the continued health-related challenges faced by countries in the African region. Despite progress, significant challenges remain in the region, including high morbidity and mortality, and poor nutrition and maternal health outcomes (Deaton and Tortora, 2015; World Health Organization, 2018). Consequently, the livelihood and economic prospects of individuals and households are reduced by these poor health outcomes as morbidity can reduce an individual’s capacity to engage in productive activities, trapping poor households in a vicious cycle of poverty, poor health, food insecurity and poor nutrition (Cai *et al.*, 2014; Nwosu and Woolard, 2017; Mazumdar *et al.*, 2014; Cai and Kalb, 2006; Dhanaraj, 2016; Novignon *et al.*, 2015). Furthermore, suboptimal investments in health during childhood can reduce cognitive abilities as well as one’s future health stock, with negative implications for future productive potential (Case and Paxson, 2008; Gertler *et al.*, 2014).

Thus, CT programmes may improve health outcomes through reduced poverty, food insecurity and productive capacity pathways. However, evidence on CTs and health outcomes is mixed. In the current literature review and analyses, we focus on healthcare utilization and morbidity while excluding related dimensions of health such as mental health, nutrition and children’s anthropometric status, as they are covered in more detail elsewhere (Manley *et al.*, 2013; Tiwari *et al.*, 2016; Manley *et al.*, 2020; Bastagli *et al.*, 2019; De Groot *et al.*, 2017; Zimmerman *et al.*, 2021). Bastagli *et al*. found that of 15 studies examining CT impacts on health facilities utilization, nine found positive impacts, one found a negative impact in Tanzania and the remaining studies found no impacts (Bastagli *et al.*, 2019). Lagarde *et al*. (2007) examined 10 articles from six studies (five of CCT programmes in Latin America and one CCT programme in Malawi) and found positive programme impacts on the use of health services in five of the six studies, although evidence on health outcomes was mixed (Lagarde *et al.*, 2007). Only some protective impacts were found on the incidence of anaemia, diarrhoea among children under 48 months and reported illness in the past month (Lagarde *et al.*, 2007). However, this latter review did not include findings from any government-run CT programmes in Africa (the sole programme from Africa provided monetary incentives for HIV testing) due to limited studies at the time of the review. Ranganathan and Lagarde (2012) examined the impacts of 13 CCT programmes (eight from Latin America and the Caribbean, two from Asia and three from Africa) on health outcomes and found that CCTs improved uptake of healthcare services in 10 programmes, but found improvements in health outcomes in only four. Similarly, in their review of CCT programmes and health outcomes, Owusu-Addo and Cross (2014) focused largely on programmes based in Latin America examining 16 publications from six studies (Owusu-Addo and Cross, 2014). The authors concluded that CCT programmes had positive impacts on health service utilization, but, consistent with the other reviews, found that evidence on morbidity was mixed. Two recent systematic reviews focusing on Africa specifically have found positive impacts on healthcare utilization but limited impacts on morbidity (Onwuchekwa *et al.*, 2021). One study reviewed eight CCTs and found that two out of three studies found positive impacts on health facility utilization, but no studies found impacts on the frequency of illness, while the other reviewed 11 UCTs, 8 CCTs and 5 combined CCT/UCTs and found that 9 out of 11 studies found positive impacts on care seeking and 7 out of 9 studies found significant impacts on child health (Owusu-Addo *et al.*, 2018). Somewhat in contrast with evidence based largely on CCTs or reviews combining CCTs and UCTs, a global meta-analysis of 21 UCTs from Africa (11 studies), Latin America (7 studies) and South-East Asia (2 studies) found that UCTs decreased the likelihood of illness and improved food security, but there was not sufficient evidence of impacts on health service utilization, despite some evidence to suggest increases in spending on healthcare (Pega *et al.*, 2017).

In summary, much of the evidence to date is biased towards CCT programmes (primarily from Latin America). Studies on the impact of UCT programmes and CTs more generally in Africa on health are limited (Owusu-Addo *et al.*, 2018). Currently, it is not possible to disentangle whether regional differences in the evidence between Latin America and Africa are driven by conditions (which are more common in Latin America) or quality of health services, as Latin America on average has stronger health systems and other contextual factors. For example, a previous study of Zambia’s CGP found no overall impacts on maternal health-seeking behaviours but did find heterogeneous impacts whereby women in communities with higher quality health facilities were more likely to have skilled attendants at birth as a result of the programme (Handa *et al.*, 2015b).

Taken together, this body of evidence suggests that CCT and UCT may have very different impacts on illness and health services utilization and that summaries of evidence on UCTs and CCTs should be disaggregated. In this study, we provide new empirical evidence on the impact of five large, government UCT programmes on morbidity and health-seeking behaviours across four countries in Africa (Ghana, Malawi, Zambia and Zimbabwe).

## Theoretical framework and pathways of impact

**Table 1.** Data summary and sample size

Programme	Districts	Sample size at baseline	Survey waves
Ghana LEAP 1000	Yendi, Karaga and East Mamprusi in the Northern Region and Bongo and Garu Tempane in the Upper East Region	2497 households (treatment = 1262; comparison = 1235)	Baseline (2015), 23 months (2017)
Malawi	Salima and Mangochi	3531 households (treatment = 1678; comparison = 1853)	Baseline (2013), 17 months (2014–15), 24 months (2015)
Zambia CGP	Shangom’bo, Kalabo and Kaputa	2515 households (treatmen t= 1228; comparison = 1287)	Baseline (2010), 24 months (2012), 36 months (2013), 48 months (2014)
Zambia MCT	Serenje and Luwingu	3078 households (treatment = 1561; comparison = 1517)	Baseline (2012), 24 months (2013), 36 months (2014)
Zimbabwe	Treatment districts: Binga, Mwenzi and Mudzi Comparison districts: UMP, Chiredzi and Hwange	3063 households (2029 treatment & 1034 comparison)	Baseline (2013), 12 months (2014)

There are several pathways through which UCTs may affect morbidity and health-seeking behaviours (De Groot *et al.*, 2017; Owusu-Addo *et al.*, 2019). For instance, food insecurity is closely linked to morbidity, and there is extensive evidence demonstrating how UCTs can improve food security, including in Africa (Bastagli *et al.*, 2019; Hidrobo *et al.*, 2018; Tiwari *et al.*, 2016).

UCTs may also reduce morbidity through an income effect and increased use of health services. Barriers to health seeking include costs related to fees, medicines, transportation and opportunity costs of lost wages. Thus, the increased economic security provided by UCTs may allow individuals to seek more preventive care including vaccines and utilize health services in a timelier manner when sick. A multi-country qualitative study found that UCTs reduced demand-side constraints in accessing healthcare for older people in four countries (Ethiopia, Tanzania, Mozambique and Zimbabwe) (Helpage International, 2017).

UCTs may also reduce stress, as poverty and food insecurity are chronic stressors. Chronic stress can have physiologically damaging effects on health through increased inflammation, dysregulation of the immune system, and cumulative wear and tear caused by continuous activation of physiological responses to stress (Aiello and Dowd, 2013; Mcewen and Seeman, 1999; Seeman and Crimmins, 2001; Steptoe and Marmot, 2002; Cohen *et al.*, 2007).

UCTs can also lead to improved sanitation (including improved water sources, purchase of soap, toilets, etc.), reducing transmission of communicable diseases. Further, following evidence that child health is influenced by mothers’ bargaining power, UCTs could improve the intra-household bargaining power of women (Bonilla *et al.*, 2017; Bastagli *et al.*, 2019), and this could positively impact women’s and children’s nutrition and health status.

Indirectly, UCTs may reduce morbidity caused by injury by reducing engagement in hazardous work. However, the empirical evidence on this relationship to date is extremely limited (De Hoop and Rosati, 2014), and one study even showed that Malawi’s Social Cash Transfer increased the likelihood of children engaging in hazardous activities (De Hoop *et al.*, 2020).

Finally, several factors at the community level may moderate the impacts of UCTs on morbidity and health-seeking behaviours. These include availability and quality of healthcare facilities (Handa *et al.*, 2015a), environmental factors such as access to clean water (Seidenfeld *et al.*, 2014; Roelen *et al.*, 2017), the infectious environment and other factors. Relatedly, where supply-side conditions are poor, interventions to incentivize healthcare demand may be harmful. One initiative to incentivize institutional delivery in India led to overcrowded health facilities with overstretched healthcare workers resulting in increased perinatal mortality rates (Andrew and Vera-Hernandez, 2020).

## Data and methods

### Data

Data used in this study came from five UCT programmes from four countries in Africa. These programmes are the Ghana Livelihood Empowerment Against Poverty (LEAP) 1000 programme, the Malawi Social Cash Transfer Programme (Malawi SCTP), the Zambia Child Grant Programme (Zambia CGP), the Zambia Multiple Categorical Targeting Programme (Zambia MCT) and the Zimbabwe National Harmonized Social Cash Transfer Programme (Zimbabwe HSCT) (American Institutes for Research, 2014a; 2014b; Ghana Leap 1000 Evaluation Team, 2018; Ward *et al.*, 2010).

Table 1 summarizes the geographical regions where data were collected, survey years and household sample sizes. In all countries examined, study designs were either cluster randomized controlled trial (RCT) (Malawi and Zambia) or quasi-experimental (Ghana and Zimbabwe). The Ghana evaluation sampled households around an eligibility score cut-off for the programme to construct a treatment and comparison group. In Zimbabwe, comparison districts were selected based on agro-ecological characteristics (they neighbour each other), culture and level of development.

We provide brief descriptions of these programmes below.

### Ghana LEAP 1000

The Ghana LEAP 1000 programme is an extension of the country’s flagship anti-poverty programme, LEAP. While LEAP is targeted to extremely poor households with orphans and vulnerable children, elderly with no productive capacity or persons with acute disability, LEAP 1000 was a pilot designed to add a new target group: pregnant women and mothers with infants under 1 year of age. The programme is implemented by the LEAP Management Secretariat (LMS) and the Department of Social Welfare, under the Ministry of Gender, Children and Social Protection. LEAP provides a bimonthly transfer, which ranges from GHc 64 to GHc 106 (approximately USD17–USD28 in 2015) based on the number of eligible beneficiaries in the household. Beneficiary households are targeted through a proxy-means test (PMT) with a sharp cut-off established by the LMS. Those households meeting the poverty criterion (a PMT score below the cut-off) were enrolled in LEAP 1000 from August 2015 onwards. In addition to the CT, eligible households received a fee waiver for enrolment into the National Health Insurance Scheme, providing access to free out-patient and in-patient services, dental services and maternal health services. The impact evaluation exploited the discontinuity at the PMT cut-off to establish a treatment and comparison group. Those falling just below the cut-off comprise the treatment group, and those just above the cut-off were sampled as the comparison group. Households close to the cut-off on both sides are highly similar in terms of their characteristics because they have very similar PMT scores. The baseline sample included 2497 households in 2015 and one follow-up was conducted after 23 months (Ghana Leap 1000 Evaluation Team, 2016; 2018).

### Malawi SCTP

In Malawi, the beneficiaries include ultra-poor and labour-constrained households for whom the programme aims to reduce poverty and hunger and increase school enrolment rates. The SCTP is operated by the Ministry of Gender, Children, Disability and Social Welfare with support from the Ministry of Finance, Economic Planning and Development as well as UNICEF Malawi. Average transfer amounts are ∼2000 Kwacha per month (∼3 USD). As of the last wave of data collection used in this study (December 2015), the programme was operating in 18 out of 28 districts and reached over 160 000 households. Households were first selected by village-level committees to identify poor households containing individuals with a chronic illness or disability and then a PMT was used to verify poverty status before households were enrolled. Transfers in the Malawi programme are adjusted for household size. There is also a schooling bonus that is based on the number of youths of school age in the household (Abdoulayi *et al.*, 2016; 2014). The evaluation utilized an RCT design with 29 village clusters randomized into treatment and delayed-entry control arms in two traditional authorities (Salima and Mangochi) and four districts (Maganga, Ndindi, Jalasi and M’bwana Nyambi). The evaluation sample consisted of 3531 eligible households at baseline in 2013, and follow-up waves were conducted at 12 months (2014) and 24 months (2015) (Abdoulayi *et al.*, 2016).

### The Zambia CGP

The Zambia CGP programme commenced in 2010 with the primary objective of reducing extreme poverty and the intergenerational transfer of poverty. Specifically, the programme sought to improve some specific health and education outcomes including (1) improvement in food security, (2) reduction in child mortality and morbidity, (3) reduction in stunting and wasting, (4) increase in school enrolment and attendance and (5) increased asset ownership (American Institutes for Research, 2011). Beneficiaries of the programme included all households with a child under age 5 years. Households with newborn babies were immediately enrolled in the programme through a continuous system. The CGP was operated by the Ministry of Community Development, Mother and Child Health in three districts (Kalabo, Kaputa and Shangombo). These districts were targeted because they face the highest rates of malnutrition, morbidity and mortality. Moreover, ∼95% of beneficiaries in the region were estimated to be living below the extreme poverty line in 2010. Beneficiaries received a flat monthly transfer, which started at 60 Zambian Kwacha (revised to 70 Zambian Kwacha in 2014; ∼11 USD) (American Institutes for Research, 2016a). The total amount translates to ∼12 Kwacha per capita per month and is estimated to be sufficient to provide one additional meal per person per day. The evaluation utilized an RCT design, with 92 communities randomized into treatment and control (delayed entry) arms in three districts (Kalabo, Kaputa and Shangombo). The evaluation sample consisted of 2515 households at baseline in 2010, and follow-up waves were conducted at 24, 30, 36 and 48 months (American Institutes for Research, 2016a).

### Zambia MCT

In Zambia, the MCT programme started in two of the most deprived districts: Luwingu and Serenje and targets: (1) households headed by widows and caring for orphans; (2) households headed by an elderly person and caring for orphans and (3) households that have a member living with some form of disability. The programme began with the broad objective of reducing poverty and the intergenerational transmission of poverty. Specifically, the programme seeks to (1) improve food security among beneficiary households, (2) increase school enrolment and attendance and (3) increase asset ownership. The programme is also operated by the Ministry of Community Development, Mother and Child Health. Flat monthly transfers are provided in the same amount as in the CGP (started at 60 Kwacha per month, revised to 70 Kwacha in 2014—∼11 USD). The evaluation utilized an RCT design, whereby 90 communities (CWACS, or community welfare assistance committees) were randomly assigned to either treatment or control (delayed entry) status, and the total evaluation sample consisted of 3078 households at baseline. Data collection for the baseline survey occurred in 2011, with 24- and 36-month follow-ups (American Institutes for Research, 2016b).

### Zimbabwe HSCT

The Zimbabwe HSCT targets labour-constrained and food-poor households and seeks to increase households’ consumption and food security. The programme is administered and managed by the Department of Social Services in the Ministry of Public Service, and funding for the programme comes from the Zimbabwe government and external donors. Transfers are distributed bi-monthly and amounts range from 10 to 25 USD per month, varying based on household size. A total of about 55 000 households were benefiting from the programme as of the last data collection utilized in this analysis (2014). The evaluation uses a case–control design, with the treatment arm drawn from three districts (Binga, Mwenzi and Mudzi) and the comparison group drawn from three neighbouring districts (UMP, Chiredzei and Hwange) matched to the treatment districts based on agro-ecological characteristics, culture and level of development. The evaluation sample consists of 3063 households in 90 wards across six districts (60 treatment wards and 30 comparison wards). Baseline data were collected in 2013, and a 12-month follow-up survey was carried out in 2014 (American Institutes for Research, 2014a).

### Measures

In this analysis, we examine the incidence of acute illness (disaggregated into specific illnesses such as fever/malaria, respiratory and diarrhoea), seeking medical care, health spending and self-assessed health (see Supplementary Appendix I for detailed indicator construction by country). Measures of child and adult health outcomes were examined separately, as information collected varied by age, except for the Zambia MCT and Zambia CGP, where the health status of children was reported with the same indicators as for all household members. In all programmes, questions were asked to the main household respondent (generally the main CT recipient or household head) on the health of all individuals in the household, with a recall period of 2–4 weeks preceding the survey (2 weeks in Ghana, Malawi and Zambia and 4 weeks Zimbabwe; Supplementary Appendix 1). Health outcomes for children under age 5 years include seeking preventive care, seeking medical treatment for various illnesses, the incidence of illness (diarrhoea, fever/malaria and cough/respiratory illness) and health spending.

Controls included age in years; sex; main respondent characteristics (age, marital status and education); household size, household access to clean water, improved toilet, the experience of shocks, per capita monthly expenditures; and district (more details and availability by country outlined in Supplementary Appendix 2). In Ghana LEAP 1000, education is measured for the household head, not the main questionnaire respondent.

### Statistical approach

We first summarize outcomes by treatment status and assess baseline balance between groups by regressing background characteristics and outcomes (at baseline) on the treatment indicator. Next, we assessed differential attrition between treatment and control groups across survey waves and age groups (sub-samples) by running a regression with an indicator for a respondent being observed at follow-up (i.e. not attritted) as the dependent variable and treatment dummy as the independent variable. A significant difference in our outcome(s) with respect to the treatment dummy in this regression would suggest that there was differential attrition, potentially threatening internal validity.

To estimate the impact of UCT programmes on morbidity and health-seeking behaviour, a difference-in-differences (DID) estimation method was used.

The estimating equation is as follows:

(1) $${h_{itj}} = {\alpha _0} + {\alpha _1}{T_i} + {\alpha _2}{R_t} + {\alpha _3}\left( {{T_i}*{R_t}} \right) + {\alpha _x}{X_i} + {\varepsilon _i}$$

where individual *i*’s treatment status is represented by *T. R* denotes the survey round taking the value of 1 for follow-up and 0 for baseline while *j* represents the health outcome of interest. *X* is a vector of baseline control variables, and $\varepsilon $ is the error term. The coefficient of the interaction term (${\alpha _3}$) indicates the intent-to-treat programme impact.

All analyses were conducted separately for each country and by age groups (under 5 years; 5–19 years; 20–59 years; 60 and above). For binary outcomes, linear probability models (LPMs) were estimated with robust standard errors adjusted for clustering at the community level. These models were chosen over logistic regressions for ease of interpretation of the interaction term (Norton *et al.*, 2004), which represents the programme effect. Moreover, LPM indicates marginal effects (percentage point changes), which are meaningful for interpretation. For continuous outcomes (health expenditures), we run ordinary least squares regressions and report coefficient estimates that can be interpreted as the average change in health expenditures in local currency.

### Data availability

Data sets for the Ghana LEAP 1000 evaluation are publicly available through the Carolina Population Center (https://data.cpc.unc.edu/projects/13/view). Other data sets are not publicly available.

## Results

Sample sizes of households ranged from 2497 in Ghana to 3531 in Malawi (Table 1). The average household size ranged from 5.65 members in Malawi to 7.69 in Ghana (Table 2). The average age of household members ranged from 15.54 years in Zambia’s CGP to 25.51 years in Malawi’s SCT. The percentage of household heads or designated CT recipients who ever attended formal schooling ranged from 18 in Ghana to 67% in Zambia’s MCT. For each country, there were no more than two imbalances in background characteristics and outcomes at baseline (Table 2), suggesting that the treatment and control/comparison groups are sufficiently balanced to attribute differences at endline to programme impacts. Baseline balance by age group is presented in Supplementary Appendix 4.

**Table 2.** Baseline means of background characteristics and outcomes at baseline (all ages), by treatment status for full baseline sample

	Panel A: Ghana LEAP 1000						
	Pooled		Comparison		Treatment		
	Mean	*n*	Mean	*n*	Mean	*n*	*P*-value
Age in years	19.360	15 512	19.577	7274	19.169	8238	0.570
Female	0.521	15 512	0.524	7274	0.519	8238	0.077
Head no formal schooling	0.819	15 512	0.799	7274	0.836	8238	0.541
Improved source of water	0.580	15 512	0.567	7274	0.591	8238	0.711
Improved source of sanitation	0.100	15 512	0.096	7274	0.104	8238	0.698
Household size	7.688	15 512	7.331	7274	8.003	8238	0.270
Total household monthly per capita expenditure (Ghana cedis)	60.710	15 512	62.516	7274	59.115	8238	0.252
Illness in last 2 weeks	0.230	11 571	0.232	5385	0.228	6186	0.846
Sought care for illness in last 2 weeks	0.546	2656	0.549	1247	0.544	1409	0.432
Real health expenditures	6.543	11 601	6.643	5401	6.455	6200	0.468
Preventive care	0.424	3521	0.421	1697	0.428	1824	0.608
Diarrhoea last 2 weeks (under 5 years)	0.391	3521	0.410	1697	0.372	1824	**0.018**
Sought care for diarrhoea (under 5 years)	0.906	1375	0.908	696	0.904	679	0.670
Fever last 2 weeks (under 5 years)	0.249	3521	0.269	1697	0.231	1824	**0.006**
Sought care for fever (under 5 years)	0.994	878	0.996	456	0.993	422	0.803
Symptoms of ARI last 2 weeks (under 5 years)	0.055	3521	0.058	1697	0.052	1824	0.743
Sought care for ARI (under 5 years)	1.000	193	1.000	99	1.000	94	
Illness in last 2 weeks (child <5 years of age)	0.572	3521	0.593	1697	0.552	1824	0.074
Sought care for illness (child <5 years of age)	0.504	2013	0.518	1007	0.490	1006	0.778
Real child health expenditures	16.041	3521	14.249	1697	17.709	1824	0.298
	**Panel B: Malawi**						
	**Pooled**		**Control**		**Treatment**		
**Variables**	**Mean**	***n***	**Mean**	***n***	**Mean**	***n***	***P*-value**
Age in years	25.51	15 251	25.04	8017	25.99	7234	0.43
Male	1.57	15 251	1.57	8017	1.57	7234	0.90
Recipient attended school	0.34	15 251	0.34	8017	0.33	7234	0.82
Household has access to some toilet facilities	0.77	15 251	0.75	8017	0.79	7234	0.45
Household has access to clean water source	0.90	15 251	0.90	8017	0.89	7234	0.87
Household was affected by any shock	0.95	15 251	0.94	8017	0.97	7234	0.57
Household size	5.65	15 251	5.65	8017	5.63	7234	0.85
Total household monthly per capita expenditure (Malawian Kwacha)	3,111.77	15 251	3,045.32	8017	3,180.38	7234	0.79
Salima district	0.42	15 251	0.45	8017	0.38	7234	0.78
Illness	0.29	15 251	0.27	8017	0.30	7234	0.08
Seek medical care	0.55	4374	0.59	2160	0.51	2214	**0.01**
Chronic illness	0.24	10 328	0.23	5382	0.26	4946	0.38
Fever/malaria	0.08	15 251	0.07	8017	0.08	7234	0.42
Respiratory	0.09	15 251	0.09	8017	0.09	7234	0.54
Diarrhaea	0.03	15 251	0.03	8017	0.03	7234	0.95
Health expenditure (Malawian Kwacha)	78.21	15 232	71.42	8005	85.21	7227	0.54
Self-assessed health	0.41	15 202	0.38	7993	0.44	7209	0.52
	**Panel C: Zambia CGP**						
	Pooled		Control		Treatment		
Variables	Mean	*n*	Mean	*n*	Mean	*n*	*P*-value
Age in years	15.54	14 156	15.36	7005	15.72	7151	0.11
Male	0.47	14 156	0.48	7005	0.47	7151	0.13
Recipient attended school	0.73	14 156	0.71	7005	0.74	7151	0.45
Household has access to some toilet facilities	0.52	14 156	0.52	7005	0.52	7151	1.00
Household has access to clean water source	0.23	14 156	0.23	7005	0.23	7151	0.94
Household was affected by any shock	0.20	14 156	0.20	7005	0.20	7151	0.91
Household size	6.48	14 156	6.36	7005	6.59	7151	0.28
Total household monthly per capita expenditure (Zambian Kwacha)	37.19	14 156	36.18	7005	38.18	7151	0.40
Kaputa district	0.36	14 156	0.36	7005	0.36	7151	0.97
Shangombo district	0.33	14 156	0.33	7005	0.33	7151	0.97
Illness	0.16	14 156	0.16	7005	0.16	7151	0.86
Seek medical care	0.70	2280	0.71	1127	0.68	1153	0.46

**Table 2.** (Continued)

Chronic illness	0.02	14 139	0.02	6996	0.02	7143	0.92
Fever/malaria	0.04	14 156	0.04	7005	0.04	7151	0.62
Respiratory	0.03	14 156	0.04	7005	0.03	7151	**0.04**
Diarrhoea	0.04	14 156	0.03	7005	0.04	7151	0.17
Health expenditure (Zambian Kwacha)	3,356.08	1850	1,943.50	922	4,759.52	928	0.16
Self-assessed health	0.47	5137	0.47	2521	0.46	2616	0.78
	**Panel D: Zambia MCT**						
	**Pooled**		**Control**		**Treatment**		
**Characteristics**	**Mean**	***n***	**Mean**	***n***	**Mean**	***n***	***P*-value**
Age in years	25.27	14 959	25.61	7411	24.95	7548	0.33
Male	0.45	14 959	0.45	7411	0.45	7548	0.72
Recipient attended school	0.67	14 959	0.68	7411	0.66	7548	0.59
Household has access to some toilet facilities	0.94	14 959	0.94	7411	0.94	7548	0.89
Household has access to clean water source	0.23	14 959	0.27	7411	0.19	7548	**0.02**
Household was affected by any shock	0.57	14 959	0.61	7411	0.53	7548	0.13
Household size	6.22	14 959	6.25	7411	6.18	7548	0.78
Total household monthly per capita expenditure (Zambian Kwacha)	42.80	14 959	43.40	7411	42.21	7548	0.61
Serenje district	0.56	14 959	0.57	7411	0.54	7548	0.79
Illness	0.15	14 959	0.16	7411	0.14	7548	0.15
Seek medical care	0.64	2180	0.64	1149	0.65	1031	0.85
Chronic illness	0.03	14 914	0.04	7399	0.03	7515	0.59
Fever/malaria	0.03	14 959	0.04	7411	0.03	7548	0.39
Respiratory	0.04	14 959	0.04	7411	0.04	7548	0.29
Diarrhoea	0.02	14 959	0.02	7411	0.02	7548	0.79
Health expenditure (Zambian Kwacha)	2,876.22	1717	3,536.99	936	2,084.31	781	0.40
Self-assessed health	0.43	6726	0.44	3391	0.42	3335	0.49
	Panel E: Zimbabwe						
	Pooled		Control		Treatment		
Characteristics	Mean	*n*	Mean	*n*	Mean	*n*	*P*-value
Age in years	25.49	14 496	25.71	4915	25.39	9581	0.76
Household size	6.56	14 496	6.57	4915	6.55	9581	0.93
Main respondent female	0.66	14 496	0.64	4915	0.67	9581	0.39
Age of main respondent	52.28	14 496	53.18	4915	51.89	9581	0.30
Main respondent widowed	0.29	14 496	0.29	4915	0.28	9581	0.68
Main respondent divorced/separated	0.09	14 496	0.07	4915	0.09	9581	0.20
Main respondent ever attended school	0.65	14 496	0.66	4915	0.64	9581	0.59
Main respondent highest grade	3.92	14 496	3.87	4915	3.95	9581	0.69
Illness	0.25	14 496	0.25	4915	0.26	9581	0.57
Seek medical care	0.70	3718	0.68	1236	0.71	2482	0.26
Chronic illness	0.10	14 491	0.09	4913	0.10	9578	0.72
Fever/malaria	0.04	14 496	0.04	4915	0.04	9581	0.39
Respiratory	0.06	14 496	0.06	4915	0.06	9581	0.68
Diarrhoea	0.03	14 496	0.03	4915	0.02	9581	0.23
Health expenditure (USD)	3.99	3718	3.97	1236	4.00	2482	0.97
Under 5 only outcomes							
Self-assessed health	0.23	14 476	0.22	4902	0.24	9574	0.28
Preventive care	0.63	1741	0.63	584	0.63	1157	0.95
Diarrhoea	0.18	1742	0.20	587	0.18	1155	0.47
Diarrhoea care	0.60	310	0.57	113	0.61	197	0.66
Fever	0.25	1742	0.27	587	0.24	1155	0.42
Fever care	0.53	589	0.54	213	0.53	376	0.78
Cough	0.36	1742	0.41	587	0.35	1155	0.05
Cough care	0.42	739	0.41	264	0.42	475	0.78

*Note*: In Ghana LEAP 1000, education is measured for the household head, not the main questionnaire respondent.

*Notes*: Bivariate regressions test difference between treatment and control groups. Self-assessed health only available for respondents 18 years and older. Standard errors are clustered at the community level. Bold denotes significance at the alpha = 0.05 level.

**Table 3.** Impact of Ghana LEAP 1000 cash transfer on morbidity and health-seeking behaviour, by age groups

Panel A: All ages (5 years and above)														
		Illness in last 2 weeks		Sought care for illness in last 2 weeks			Fever last 2 weeks		Symptoms of ARI last 2 weeks		Diarrhoea last 2 weeks		Real health expenditures	
Impact		0.01		0.04									−0.89	
		(0.01)		(0.03)									(1.02)	
*R* ^2^		0.07		0.09									0.02	
*n*		27 556		7585									27 779	
**Panel B: Children 5–19 years**														
Impact		−0.00			−0.04								−0.41	
		(0.02)			(0.05)								(0.91)	
*R* ^2^		0.07			0.17								0.03	
*n*		10 244			1731								10 260	
**Panel C: Adults 20–59 years**														
Impact		0.01			0.11***								1.00	
		(0.02)			(0.04)								(1.40)	
*R* ^2^		0.08			0.14								0.02	
*n*		10 147			2279								10 180	
**Panel D: Adults 60+ years**														
Impact		−0.02			0.01								−7.99	
		(0.04)			(0.15)								(4.96)	
*R* ^2^		0.13			0.39								0.16	
*n*		1393			270								1393	
**Panel E: Children under age 5 years**														
	**Preventive care**		**Diarrhoea last 2 weeks**		**Sought care for** **diarrhoea**	**Fever last 2 weeks**		**Sought care for fever**		**Symptoms of ARI last 2 weeks**		**Sought care for ARI**		**Real health expenditures**
Impact	0.02		0.03		−0.01	0.05**		0.03		−0.00		0.12		−4.25*
	(0.03)		(0.03)		(0.03)	(0.02)		(0.02)		(0.01)		(0.07)		(2.51)
*R* ^2^	0.08		0.09		0.15	0.08		0.13		0.04		0.31		0.04
*n*	5771		5771		2076	5771		1502		5771		237		5771

*Note*: The above impact estimates are obtained using DID estimations with ordinary least square regressions. Panel A presents estimates for all ages while the subsequent panels are disaggregated by age groups. For Ghana LEAP 1000, the prevalence of the illnesses has different denominators because questions on fever, diarrhoea and ARI were only asked to children under 5 years. All regressions control for individual characteristics including age and sex along with household characteristics including household size, whether the head attended any school, access to clean water, access to a toilet, per capita monthly expenditures and community fixed effects. Incidence of diarrhoea, fever and symptoms of ARI only available for children under 5 years. Standard errors are clustered at the community level. Robust t-statistics in parentheses.

* *P *< 0.1.

** *P *< 0.05.

*** *P *< 0.01.

**Table 4.** Impact of Malawi SCT cash transfer on morbidity and health-seeking behaviour, by age groups

	Illness	Seek medical care	Chronic illness	Fever/malaria	Respiratory	Diarrhoea	Health expenditure	Self-assessed health
**Panel A: All ages**								
17 months	0.066***	0.079**	−0.028	−0.012	−0.010	−0.005	0.113	−0.085
	(2.77)	(2.34)	(1.21)	(1.31)	(1.26)	(0.85)	(0.38)	(0.80)
*R* ^2^	0.08	0.02	0.30	0.01	0.03	0.00	0.07	0.09
*n*	28 451	7292	19 853	28 451	28 451	28 451	28 407	28 369
24 months	−0.048	0.081**	−0.031	−0.031***	−0.008	−0.005	−0.076	−0.007
	(1.65)	(2.17)	(1.19)	(3.03)	(0.69)	(0.94)	(0.26)	(0.07)
*R* ^2^	0.08	0.02	0.27	0.01	0.03	0.00	0.07	0.08
*n*	28 519	7875	20 424	28 519	28 519	28 519	28 500	28 470
**Panel B: Ages 5–19 years**								
17 months	−0.037*	0.132**	−0.017	−0.010	−0.001	−0.005	0.223	
	(1.93)	(2.34)	(1.68)	(1.15)	(0.13)	(0.98)	(1.01)	
*R* ^2^	0.01	0.03	0.01	0.01	0.01	0.00	0.02	
*n*	14 902	2367	9773	14 902	14 902	14 902	14 879	
24 months	−0.029	0.102**	−0.013	−0.020*	−0.002	−0.012***	0.148	
	(1.29)	(2.07)	(0.86)	(1.74)	(0.18)	(2.96)	(0.63)	
*R* ^2^	0.00	0.03	0.00	0.01	0.01	0.00	0.02	
*n*	14 951	2607	10 369	14 951	14 951	14 951	14 941	
**Panel C: Ages 20–59 years**								
17 months	−0.070	0.097*	−0.063*	−0.023	0.014	−0.015	0.127	−0.077
	(1.40)	(1.74)	(1.80)	(1.10)	(0.96)	(1.56)	(0.30)	(0.76)
*R* ^2^	0.06	0.02	0.09	0.03	0.02	0.01	0.06	0.05
*n*	5383	1484	5381	5383	5383	5383	5375	5369
24 months	−0.074	0.114**	−0.064*	−0.068***	0.010	−0.008	−0.460	0.005
	(1.56)	(2.10)	(1.83)	(3.95)	(0.56)	(0.83)	(1.04)	(0.04)
*R* ^2^	0.05	0.02	0.07	0.03	0.02	0.01	0.05	0.04
*n*	5416	1683	5414	5416	5416	5416	5412	5408
**Panel D: Ages 60 plus years**								
17 months	−0.130***	0.018	−0.016	0.002	−0.044	−0.011	−0.143	−0.025
	(3.09)	(0.35)	(0.24)	(0.10)	(1.65)	(0.58)	(0.27)	(0.41)
*R* ^2^	0.07	0.03	0.05	0.02	0.03	0.01	0.05	0.03
*n*	4696	2286	4693	4696	4696	4696	4690	4677
24 months	−0.115**	0.019	−0.033	−0.048**	−0.044	0.005	−0.687	0.029
	(2.49)	(0.34)	(0.48)	(2.38)	(1.54)	(0.26)	(1.18)	(0.47)
*R* ^2^	0.04	0.03	0.04	0.02	0.03	0.00	0.03	0.02
*n*	4633	2393	4628	4633	4633	4633	4631	4621
**Panel E: Children under age 5 years**								
	**Preventive care**	**Diarrhoea**	**Diarrhoea care**	**Fever**	**Fever care**	**Cough**	**Cough care**	**Health expenditure**
17 months	0.037	0.016	0.012	0.013	0.164*	−0.001	0.030	0.083
	(0.43)	(0.72)	(0.15)	(0.44)	(1.99)	(0.02)	(0.33)	(0.23)
*R* ^2^	0.16	0.07	0.06	0.04	0.05	0.05	0.03	0.04
*n*	3185	3185	418	3185	752	3185	609	3463
24 months	0.007	−0.009	0.255**	0.030	0.157	0.048	−0.003	0.329
	(0.07)	(0.36)	(2.13)	(0.67)	(1.49)	(0.99)	(0.03)	(0.84)
*R* ^2^	0.13	0.05	0.07	0.02	0.04	0.04	0.05	0.04
*n*	2824	2824	400	2824	695	2824	599	3516

*Note*: The above impact estimates are obtained using DID estimations with ordinary least square regressions. Panel A presents estimates for all ages while the subsequent panels are disaggregated by age groups. Self-assessed health only available for ages 5+ years. All regressions control for individual characteristics including age, sex and whether the individual attended any school along with household characteristics including household size, access to clean water, access to a toilet, whether the household experienced any shocks, per capita monthly expenditures and district fixed effects. Standard errors are clustered at the community level. Robust t-statistics in parentheses.

* *P *< 0.1.

** *P *< 0.05.

*** *P *< 0.01.

In terms of health outcomes, the percentage of household members reporting an illness in the previous 2–4 weeks ranged from 15% in Zambia’s MCT to 29% in Malawi. Among those who were ill, the percentage reporting health services utilization ranged from 55% in Ghana and Malawi to 70% in Zimbabwe. Looking at specific illnesses, the percentage of individuals reporting a fever in the recall period ranged from 3% in Zambia MCT to 8% in Malawi (or 25% among children under 5 years in Ghana), and the percentage reporting respiratory illness ranged from 3% in Zambia CGP to 9% in Malawi.

Available reports on the various programmes suggest that overall attrition in the surveys was minimal. For instance, in Ghana, >93% of the baseline sample was followed up after 2 years (Ghana Leap 1000 Evaluation Team, 2018). In the Zambia CGP, >96% of households from baseline remained in the 36-month and 48-month follow-ups, respectively (American Institutes for Research, 2015a; 2015b). Similar statistics were observed for the Zambia MCT(American Institutes for Research, 2016b). In Zimbabwe, ∼86% of households from baseline remained in the 12-month follow-up (American Institutes for Research, 2014a).

The attrition results in Supplementary Appendix 3 suggest there is differential attrition between treatment arms in the Zambia MCP, but not in any of the other samples. We further explored differences in baseline balance in outcomes and characteristics by treatment status among each age group in the panel sample (i.e. those who were not lost to follow-up 4) in the Zambia MCT in Supplementary Appendix 4 and find a balance between the panel sample across treatment arms, indicating that baseline balance between treatment arms was maintained in the panel sample and supporting internal validity of our findings.

In this section, we present programme impact estimates across countries, for the panel samples by age groups. For the full sample, we find no impacts of the Ghana LEAP 1000 programme on the incidence of illness, healthcare seeking and health expenditures (Table 3). Among the subgroups, we observe no impacts for the group of children 5–19 years and the elders aged 60 years and older. Among adults aged 20–59 years, we find a significant positive impact on seeking healthcare when ill (11 pp). For children under 5 years, there is an increase in the incidence of fever (5 pp) and a decrease in health expenditures ($\hat \beta $=−4.25 cedis [USD 0.97]).

Turning to the Malawi SCTP (Table 4), we find relatively more consistent impacts on health seeking and to some extent, morbidity. For example, we find a positive programme impact on health seeking at both the 17-month and 24-month follow-ups (7.9 pp and 8.1 pp, respectively), and this impact (ranges from 9.7 pp to 13.2 pp) is found among 5 to 59 age groups. There were no similar impacts on health seeking for adults aged 60 and older. Among all individuals above age 5 years, we see that incidence of self-reported fever/malaria was reduced by the programme but only at 24 months (−3.1 pp). We also find protective effects against overall morbidity (illness; Column 1) at 17 but not 24 months (−6.6 pp), which appears to be largely driven by those aged 60 years and older (−13 pp). Morbidity was also reduced by 11.5 pp for this age group at 24 months, although no overall reduction was found in the pooled sample. We further find protective effects against chronic illness (6.3–6.4 pp) at both follow-up rounds among those aged 20–59 years (but not among any other age group). Among children under age 5 years, we see no protective impacts on incidence of illness, but we do find positive programme impacts on health seeking when the child had diarrhoea (25.5 pp at 24 months) and fever (16.4 pp).

In Table 5, we examine the impacts of the Zambia MCT and find mixed results. First, we find positive programme impacts on health expenditures (reported at the individual level for those who report illness) at both waves [at 24 months increases of 1.081 ZMW (0.17 USD) and 1.371 ZMW (0.22 USD) among all ages and 5–19-year-olds, respectively, and at 36 months, an increase of 0.864 ZMW (0.14 USD) among all ages]. Contrary to our hypotheses that CTs can reduce morbidity, we found that the Zambia MCT increased reported illness at 24 months in both children under age 5 years and adults aged 60 years and above (7.6 pp and 6.6 pp, respectively). Additionally, we find that the programme increased reports of fever/malaria among children under 5 years at 24 months (4.7 pp), chronic illness among adults aged 20–59 years at 24 months (1.7 pp) and respiratory illness among those aged 60 years and over at 24 months (4.1 pp). However, these adverse impacts disappear at 36-month follow-up. We also found small, but statistically significant protective impacts against chronic illness (−0.6 pp) and fever/malaria (−1.3 pp) among children and adolescents aged 5–19 years at the 36-month follow-up.

**Table 5.** Impact of Zambia MCT cash transfer on morbidity and health-seeking behaviour, by age groups

	Illness	Seek medical care	Chronic illness	Fever/malaria	Respiratory	Diarrhoea	Health expenditure	Self-assessed health
**Panel A: All ages**								
24 months	0.021	−0.007	0.007	0.001	0.012**	0.001	1.081**	0.023
	(1.45)	(0.17)	(1.17)	(0.17)	(2.15)	(0.30)	(2.11)	(0.55)
*R* ^2^	0.04	0.02	0.04	0.01	0.01	0.00	0.05	0.23
*n*	28 966	3547	28 906	28 966	28 966	28 966	2847	13 522
36 months	0.008	−0.068	0.002	−0.001	0.002	−0.002	0.864*	0.053
	(0.62)	(1.61)	(0.31)	(0.25)	(0.30)	(0.57)	(1.68)	(1.16)
*R* ^2^	0.04	0.03	0.04	0.00	0.02	0.00	0.05	0.26
*n*	29 082	3754	29 004	29 082	29 082	29 082	3067	13 754
**Panel B: Children under 5 years**								
24 months	0.076**	−0.018	0.005	0.030	0.023	−0.020	1.079	
	(2.01)	(0.23)	(0.98)	(1.28)	(1.04)	(0.83)	(1.30)	
*R* ^2^	0.02	0.01	0.01	0.03	0.02	0.01	0.04	
*n*	2603	564	2590	2603	2603	2603	489	
36 months	0.066	−0.041	0.003	0.047*	0.027	−0.030	0.362	
	(1.49)	(0.48)	(0.46)	(1.81)	(1.10)	(1.32)	(0.50)	
*R* ^2^	0.02	0.03	0.00	0.02	0.02	0.01	0.06	
*n*	2467	524	2449	2467	2467	2467	453	
**Panel C: Ages 5–19 years**								
24 months	0.016	−0.000	−0.003	−0.004	0.008	0.005	1.371*	
	(1.24)	(0.01)	(0.94)	(0.60)	(1.37)	(1.13)	(1.85)	
*R* ^2^	0.01	0.02	0.00	0.01	0.01	0.00	0.11	
*n*	14 333	1140	14 304	14 333	14 333	14 333	971	
36 months	−0.003	−0.078	−0.006*	−0.013*	−0.001	0.001	1.046	
	(0.21)	(1.12)	(1.81)	(1.87)	(0.13)	(0.31)	(1.34)	
*R* ^2^	0.01	0.03	0.00	0.01	0.01	0.00	0.12	
*n*	14 430	1239	14 385	14 430	14 430	14 430	1069	
**Panel D: Ages 20–59 years**								
24 months	−0.010	−0.032	0.017*	−0.009	0.001	0.003	1.246	0.003
	(0.57)	(0.45)	(1.78)	(1.28)	(0.16)	(0.67)	(1.51)	(0.05)
*R* ^2^	0.04	0.03	0.02	0.01	0.02	0.01	0.06	0.09
*n*	7942	837	7929	7942	7942	7942	667	7883
36 months	−0.014	−0.079	0.013	−0.008	−0.005	−0.000	1.162	0.060
	(0.83)	(1.18)	(1.24)	(1.13)	(0.57)	(0.03)	(1.44)	(1.02)
*R* ^2^	0.04	0.04	0.03	0.00	0.01	0.00	0.06	0.11
*n*	8083	862	8073	8083	8083	8083	698	8034
**Panel E: Ages 60 plus years**								
24 months	0.066*	0.019	0.023	0.015	0.041**	0.002	0.728	0.048
	(1.77)	(0.32)	(0.85)	(1.24)	(2.63)	(0.18)	(1.25)	(1.49)
*R* ^2^	0.05	0.04	0.03	0.01	0.02	0.00	0.03	0.04
*n*	4088	1006	4083	4088	4088	4088	720	4072
36 months	0.052	−0.061	0.004	0.019	0.014	−0.005	0.676	0.036
	(1.40)	(1.06)	(0.15)	(1.33)	(0.81)	(0.42)	(1.22)	(1.42)
*R* ^2^	0.04	0.03	0.03	0.01	0.01	0.00	0.02	0.04
*n*	4102	1129	4097	4102	4102	4102	847	4090

*Note*: The above impact estimates are obtained using DID estimations with ordinary least square regressions. Panel A presents estimates for all ages while the subsequent panels are disaggregated by age groups. Self-assessed health only available for ages 18+ years . All regressions control for individual characteristics including age, sex and whether the individual attended any school along with household characteristics including household size, access to clean water, access to a toilet, whether the household experienced any shocks, per capita monthly expenditures and district fixed effects. Standard errors are clustered at the community level. Robust t-statistics in parentheses.

* *P *< 0.1.

** *P *< 0.05.

In Table 6, we examine the second programme in Zambia, the CGP, and find evidence of protective programme impacts against diarrhoea at 24 months (−1.2 pp) and 48 months (−0.9 pp) in the full sample, and among those aged 20–59 years (−1.2 pp) and under 5 years (−4.7 pp) at 24 months. We also find positive impacts on preventive care among those under 5 years at 48 months (6.8 pp) and on health seeking when ill at 36 months among those aged 20–59 years (12.9 pp). Contrary to our hypotheses, we found that the CGP increased reports of respiratory illness in the full sample at 48 months (1.1 pp) and among those aged 5–19 years at 24 months (1.4 pp). The latter, however, disappears in subsequent follow-ups.

**Table 6.** Impact of Zambia CGP cash transfer on morbidity and health-seeking behaviour, by age groups

	Illness	Seek medical care	Chronic illness	Fever/malaria	Respiratory	Diarrhoea	Health expenditure	Self-assessed health
**Panel A: All ages**								
24 months	−0.016	0.025	−0.002	−0.001	0.007	−0.012**	0.101	0.063
	(1.08)	(0.60)	(0.51)	(0.12)	(1.45)	(2.22)	(0.19)	(1.43)
*R* ^2^	0.02	0.03	0.01	0.02	0.00	0.01	0.08	0.19
*n*	27 309	3921	27 288	27 309	27 309	27 309	3347	9878
36 months	−0.010	0.061	−0.000	−0.006	0.006	−0.006	−0.187	−0.020
	(0.75)	(1.24)	(0.08)	(0.87)	(1.29)	(1.21)	(0.33)	(0.54)
*R* ^2^	0.03	0.02	0.02	0.01	0.01	0.01	0.05	0.18
*n*	29 136	3562	29 113	29 136	29 136	29 136	2962	10 490
48 months	−0.006	0.033	−0.000	0.001	0.011**	−0.009*	0.067	
	(0.41)	(0.86)	(0.11)	(0.14)	(2.12)	(1.77)	(0.13)	
*R* ^2^	0.01	0.02	0.01	0.01	0.00	0.01	0.06	
*n*	28 927	3962	28 906	28 927	28 927	28 927	3336	
**Panel B: Ages 5–19 years**								
24 months	0.011	−0.036	−0.003	0.006	0.014**	−0.006	0.128	
	(0.74)	(0.50)	(0.96)	(0.76)	(2.47)	(1.06)	(0.17)	
*R* ^2^	0.01	0.04	0.00	0.01	0.00	0.00	0.09	
*n*	10 499	1039	10 492	10 499	10 499	10 499	896	
36 months	−0.007	−0.097	−0.001	−0.000	0.007	−0.008	0.134	
	(0.54)	(1.37)	(0.21)	(0.07)	(1.32)	(1.53)	(0.17)	
*R* ^2^	0.01	0.04	0.01	0.01	0.01	0.00	0.07	
*n*	11 638	923	11 629	11 638	11 638	11 638	775	
48 months	0.001	−0.063	−0.003	0.014	0.008	−0.011*	0.393	
	(0.06)	(0.98)	(0.67)	(1.48)	(1.42)	(1.82)	(0.54)	
*R* ^2^	0.01	0.04	0.00	0.01	0.01	0.00	0.07	
*n*	12 191	1169	12 183	12 191	12 191	12 191	1008	
**Panel C: Ages 20–59 years**								
24 months	−0.027	−0.041	−0.005	−0.008	0.007	−0.012*	1.044	0.071
	(1.27)	(0.69)	(0.67)	(1.17)	(1.02)	(1.75)	(1.48)	(1.54)
*R* ^2^	0.03	0.04	0.02	0.01	0.01	0.01	0.09	0.18
*n*	8815	1044	8813	8815	8815	8815	849	8791
36 months	−0.001	0.129*	−0.004	0.005	0.005	−0.001	−0.115	−0.022
	(0.07)	(1.81)	(0.48)	(0.70)	(0.78)	(0.19)	(0.15)	(0.57)
*R* ^2^	0.04	0.03	0.02	0.01	0.01	0.01	0.05	0.17
*n*	9340	949	9338	9340	9340	9340	737	9315
48 months	−0.009	0.076	0.000	0.007	0.003	−0.004	0.558	
	(0.46)	(1.15)	(0.02)	(1.00)	(0.39)	(0.70)	(0.76)	
*R* ^2^	0.03	0.02	0.02	0.01	0.01	0.01	0.04	
*n*	9255	1063	9252	9255	9255	9255	846	
**Panel D: Ages 60 plus years**								
24 months	−0.033	0.108	0.037	0.007	0.029	−0.028	4.344*	−0.011
	(0.30)	(0.45)	(0.50)	(0.27)	(0.81)	(0.53)	(1.91)	(0.11)
*R* ^2^	0.14	0.11	0.09	0.04	0.06	0.07	0.37	0.12
*n*	352	76	351	352	352	352	63	349
36 months	0.077	−0.022	0.020	0.020	0.006	−0.005	4.839	0.002
	(0.84)	(0.10)	(0.32)	(0.69)	(0.17)	(0.13)	(1.49)	(0.03)
*R* ^2^	0.16	0.30	0.11	0.02	0.05	0.08	0.30	0.13
*n*	412	61	411	412	412	412	50	409
48 months	0.046	0.379**	0.065	−0.033	0.037	−0.018	5.125*	
	(0.50)	(2.06)	(0.97)	(0.86)	(0.93)	(0.43)	(1.98)	
*R* ^2^	0.13	0.22	0.11	0.03	0.04	0.06	0.24	
*n*	431	92	430	431	431	431	76	
**Panel E: Children under age 5 years**								
	**Preventive care**	**Diarrhoea**	**Diarrhoea care**	**Fever**	**Fever care**	**Cough**	**Cough care**	**Health expenditure**
24 months	−0.030	−0.047**	0.015	−0.012	0.007	−0.012	−0.025	−0.679
	(0.80)	(2.00)	(0.26)	(0.35)	(0.11)	(0.35)	(0.88)	(1.23)
*R* ^2^	0.03	0.05	0.02	0.04	0.04	0.04	0.06	0.08
*n*	7391	7407	1047	7433	1352	7433	7435	1533

**Table 6.** (Continued)

36 months	−0.003	−0.016	0.141	0.003	0.013	0.003	−0.005	−0.577
	(0.09)	(0.73)	(1.45)	(0.08)	(0.17)	(0.08)	(0.17)	(1.02)
*R* ^2^	0.05	0.08	0.02	0.07	0.03	0.07	0.10	0.06
*n*	7488	7473	901	7499	1234	7499	7498	1400
48 months	0.068*	−0.024	0.017	0.123	0.039	0.123	0.017	−0.591
	(1.69)	(0.93)	(0.28)	(0.67)	(0.67)	(0.67)	(0.53)	(1.10)
*R* ^2^	0.04	0.04	0.02	0.00	0.03	0.00	0.06	0.07
*n*	6810	6801	991	6827	1320	6827	6824	1403

*Note*: The above impact estimates are obtained using DID estimations with ordinary least square regressions. Panel A presents estimates for all ages while the subsequent panels are disaggregated by age groups. Self-assessed health only available for ages 18+ years . All regressions control for individual characteristics including age sex, and whether the individual attended any school along with household characteristics including household size, access to clean water, access to a toilet, whether the household experienced any shocks, per capita monthly expenditures and district fixed effects. Standard errors are clustered at the community level. Robust t-statistics in parentheses.

* *P *< 0.1.

** *P *< 0.05.

Next, in Table 7, we examine estimates from the Zimbabwe HSCT, where we largely find no impacts on morbidity or health seeking. However, we do find a small but positive impact on health expenditures (0.423 dollars) for individuals aged 60 years and above reporting an illness. Finally, contrary to our hypothesis, there was a positive and significant programme impact on diarrhoea incidence among the 20–59 age sample (1.6 pp).

**Table 7.** Impact of Zimbabwe HSCT cash transfer on morbidity and health-seeking behaviour, by age groups

	Illness	Seek medical care	Chronic illness	Fever/malaria	Respiratory	Diarrhoea	Health expenditure	Self-assessed health
**Panel A: All ages**								
12 months	−0.001	−0.026	−0.001	0.003	−0.020	0.011	−0.107	0.026
	(0.06)	(0.57)	(0.08)	(0.26)	(1.33)	(1.55)	(0.52)	(1.11)
*R* ^2^	0.11	0.03	0.11	0.04	0.02	0.00	0.08	0.22
*n*	27 168	7125	27 163	27 168	27 168	27 168	7125	27 148
**Panel B: Ages 5–19 years**								
12 months	−0.010	−0.096	−0.006	0.001	−0.019	0.005	−0.662	0.024
	(0.40)	(1.31)	(1.12)	(0.06)	(1.09)	(0.70)	(1.60)	(1.06)
*R* ^2^	0.03	0.08	0.01	0.04	0.02	0.00	0.15	0.02
*n*	13 280	2233	13 276	13 280	13 280	13 280	2233	13 267
**Panel C: Ages 20–59 years**								
12 months	0.003	0.008	0.015	0.011	−0.021	0.016**	−0.556	0.036
	(0.12)	(0.12)	(0.73)	(0.84)	(1.18)	(2.54)	(1.58)	(1.09)
*R* ^2^	0.08	0.05	0.05	0.04	0.03	0.01	0.06	0.07
*n*	5715	1498	5714	5715	5715	5715	1498	5713
**Panel D: Ages 60 plus years**								
12 months	0.011	0.067	−0.006	0.006	−0.036	0.018	0.423*	0.039
	(0.30)	(1.29)	(0.22)	(0.48)	(1.48)	(1.08)	(1.73)	(1.06)
*R* ^2^	0.08	0.02	0.04	0.04	0.01	0.01	0.06	0.08
*n*	5016	2616	5016	5016	5016	5016	2616	5014
**Panel E: Children under age 5 years**								
	**Preventive care**	**Diarrhoea**	**Diarrheoa care**	**Fever**	**Fever care**	**Cough**	**Cough care**	**Health expenditure**
12 months	0.010	0.044	0.042	0.070	−0.059	0.081	0.024	0.244
	(0.25)	(1.21)	(0.34)	(1.35)	(0.76)	(1.60)	(0.30)	(0.74)
*R* ^2^	0.13	0.05	0.10	0.05	0.10	0.04	0.08	0.16
*n*	3103	3104	504	3104	941	3104	1245	778

*Note*: The above impact estimates are obtained using DID estimations with ordinary least square regressions. Panel A presents estimates for all ages while the subsequent panels are disaggregated by age groups. All regressions control for individual characteristics including age and sex and characteristics of the main respondent including age, sex, marital status and schooling along with household characteristics including household size, access to clean water and district fixed effects. Standard errors are clustered at the community level. Robust t-statistics in parentheses.

* *P *< 0.1.

** *P *< 0.05.

## Discussion

We examine the impacts of five government-run UCT programmes on morbidity and health-seeking behaviour in four African countries. The findings indicate that the programmes have strong positive impacts on health seeking when ill in Malawi and among some age groups in Zambia and Ghana, but not in Zimbabwe. However, we find limited protective impacts on morbidity, with significant variation by age group, across all programmes.

Among children, one indicator of morbidity where we did find protective impacts was diarrhoea in Malawi and Zambia CGP. Diarrhoea is one of many complex determinants of child nutritional status, and studies of individual CTs to date have found mixed impacts on improving nutritional status (e.g. reducing stunting) (Manley *et al.*, 2013; De Groot *et al.*, 2017), although a recent meta-analysis did find positive impacts on linear growth and reductions in stunting (Manley *et al.*, 2020). Combined with results from previous studies finding positive impacts of CTs on food security (Hidrobo *et al.*, 2018), our analysis suggests that UCTs may be able to influence some of these causal pathways contributing to nutritional status.

Overall, the findings are generally consistent with existing literature on the impact of CTs on health outcomes, including findings that CCT programmes across various countries also demonstrated positive impacts on health-seeking behaviours (Ranganathan and Lagarde, 2012; Onwuchekwa *et al.*, 2021). However, a review of UCTs only found that UCTs may not have impacted the likelihood of health services utilization but that they did have a large reduction impact on morbidity in the previous 2 weeks to 3 months (Pega *et al.*, 2017). In contrast with that study, we find positive impacts on health services utilization. Aligned with that study, however, we find some impacts on morbidity reduction, but our impacts were mixed across countries and illness studied, and we did find some unexpected, adverse impacts on morbidity. The absence of programme impacts on some morbidity outcomes has been reported in previous studies (Robertson *et al.*, 2013; Onwuchekwa *et al.*, 2021; Owusu-Addo and Cross, 2014).

Several programme-related factors could influence the magnitude and breadth of programme impacts, including transfer size, payment regularity and targeting criteria. The size of these transfers at baseline ranged from 16% of pre-programme household monthly expenditures in Ghana’s LEAP 1000 to 27% in the Zambia CGP. More transformational effects are generally seen when transfers are at least 20% of pre-programme expenditures (Davis and Handa, 2015). However, this does not appear to be the case for outcomes studied here, as the country with findings most consistent with our hypotheses and across multiple outcomes was the Malawi SCTP (transfers were 17% of pre-programme expenditures), where protective impacts were observed for several illnesses and health-seeking behaviours, but not expenditures. We saw impacts across all the age groups, although among children under age 5 years improvements were seen only in care seeking. However, Malawi did have the smallest average household size among countries examined, and thus transfers may go further in smaller households. Among the programmes studied, payments were regularly made on time during the periods studied. One aspect where the programmes did vary significantly was targeting criteria. Ghana’s LEAP 1000 and Zambia’s CGP targeted households with young children and thus were more likely to have able-bodied adults of reproductive age. Members of these households might be on average healthier, but children have more preventive healthcare needs (e.g. vaccinations, etc.). Conversely, Malawi’s SCT, Zambia’s MCT and Zimbabwe’s HSCT targeted labour-constrained households (those less likely to have healthy, working-age adults), and these households might have more healthcare needs related to morbidity.

Contextual factors not studied here may also influence CT impacts on health. While income effects of CTs can increase food security and ability to pay for healthcare and can lead to some preventative behaviours (e.g. shoe ownership that can prevent some infectious diseases like exposure to helminths; Evans *et al.*, 2017; Mascarini-Serra, 2011), other environmental factors may be more difficult to overcome with cash alone. These may include infectious environments, household- and community-level practices related to hygiene and sanitation, or supply-side barriers to quality healthcare. Some of these factors may need additional efforts in the form of ‘cash plus’, defined as complementary programming such as behaviour change communication or linkages to other services, to bolster the effects of cash (Roelen *et al.*, 2017). Sun *et al*. (2020) noted variations in populations, social conditions and mechanisms may complicate the relationship between CTs and health (Sun *et al.*, 2020). This is also confirmed by evidence from a systematic review that showed that factors relating to intervention design, macro-economic stability, household dynamics and community acceptance may influence programme effectiveness (Owusu-Addo *et al.*, 2018).

Duration of follow-up periods may also influence findings, as some follow-up periods may be too short to realize impacts on morbidity and health-seeking behaviours. For example, a study of a CCT in Tanzania found that impacts on sick days only materialized after 2.5 years, and none were observed after only 1.5 years (Evans *et al.*, 2017). The lack of programme impacts for the Zimbabwe HSCT may be attributed to the fact that data were only available after 12 months. Also in Zambia’s CGP, some impacts on health-seeking (among children under 5 years and adults over 60 years) were only observed at the 48-month (but not 24 or 36 months) follow-up. This combined evidence suggests that protective impacts on morbidity may take a while to materialize, working through pathways such as improved sanitation. Indeed, as reported elsewhere, the CGP positively affected households’ likelihood of having a toilet and a cement floor (American Institutes for Research, 2016a), and we find reductions in diarrhoea incidence at both 24 and 48 months. Thus, a lack of immediate programme impacts should not be seen as ineffectiveness or reason for programme discontinuation. Moreover, others have argued that because CTs address fundamental causes and effects of distal health outcomes over time, we need to take a longer-term (and inter-generational) approach to evaluate CT impacts on health (Sun *et al.*, 2020).

The findings show that the UCT programmes in Zambia improved health outcomes and health-seeking behaviours, but impacts differed slightly across the two programmes. For instance, while both programmes showed some impact on health outcomes and health expenditures, only the Zambia CGP showed programme impacts on care seeking. Differences may be driven by the demographics of the households targeted. The MCT targeted labour-constrained households with older average age of household members. Conversely, the CGP targeted households with young children. Indeed, for children under 5 years, the Zambia CGP showed programme impacts on preventive healthcare and diarrhoea at different waves.

There are some limitations to our study. The outcome variables used were not consistent across all countries, and thus, our ability to compare across countries for some outcomes is limited. Our period of follow-up also varied across countries, ranging from 12 months in Zimbabwe to 48 months in Zambia’s CGP. This may limit the extent of observed impact—if certain impacts take longer to materialize—and hence comparability. Future studies should use longer follow-up periods if possible. The study design varied across studies, with some being RCTs (Malawi, Zambia) and others quasi-experimental (Ghana and Zimbabwe). The case for internal validity, while satisfied in all the included studies based on baseline equivalence between study arms, is stronger in RCTs. We were unable to test the parallel trends assumption for DID models in our studies because we have only one data point before programme enrolment (i.e. the baseline survey round). This is typical of impact evaluations, where budget limitations preclude multiple surveys being fielded before programme roll-out. Finally, in a majority of the villages surveyed, these national CT programmes were the only CT operating, especially in remote areas. However, we cannot rule out the possibility that some NGO-run CTs were simultaneously being run in a small number of localities, but the probability of this and that the coverage would be overlapping is so small that we do not believe it affects our findings.

This study fills an important gap, as several previous reviews on CTs and health have been heavily weighted towards programs from Latin America or CCTs (Lagarde *et al.*, 2007; Owusu-Addo and Cross, 2014; Ranganathan and Lagarde, 2012). There are major contextual differences, including levels of poverty, availability of services and infectious disease burden, as well as programmatic differences, such as health-related conditions, modality of transfers and targeting, between regions that warrant more evidence from Africa. Our findings show that UCT programmes can empower poor households to seek care when ill. However, given mixed findings on morbidity, there may be a need to simultaneously strengthen supply-side efforts to improve service availability and readiness, but more research is still needed to understand why these UCTs have had a greater impact on care seeking over reducing morbidity and the mechanisms of impact.

## Conclusion

This study evaluated the impact of UCT programmes on health and health-seeking behaviours across five UCT programmes in four countries in Africa. Findings indicate stronger impacts on health services utilization, but while positive programme impacts were observed on selected morbidity indicators, this was not consistent across all sub-samples and survey waves. More research is needed on longer-term impacts, mechanisms of impact and moderating factors such as service readiness and availability, among others. Moreover, our findings, combined with existing evidence, suggest the need for disaggregating findings on UCT and CCT when summarizing evidence on health.

## Supplementary Material

czac014_Supp

## LEAP 1000 evaluation team

UNICEF Office of Research—Innocenti: Tia Palermo (co-Principal Investigator), Richard de Groot and Elsa Valli; Institute of Statistical, Social and Economic Research, University of Ghana: Isaac Osei-Akoto (co-Principal Investigator), Clement Adamba, Joseph K. Darko, Robert Darko Osei, Francis Dompae and Nana Yaw; Carolina Population Center, University of North Carolina at Chapel Hill: Sudhanshu Handa(co-Principal Investigator), Clare Barrington (co-Principal Investigator), Sara Abdoulayi, Gustavo Angeles, Averi Chakrabarti and Frank Otchere; Navrongo Health Research Centre: Akalpa J. Akaligaung (Principal Investigator) and Raymond Aborigo.

## Zambia ZGP evaluation team

American Institutes for Research: David Seidenfeld (Principal Investigator), Juan Bonilla, Alvaro Ballarin Cabrera, Thomas De Hoop, Leah Prencipe, Nisha Rai, Hannah Reeves, Dan Sherman, Jonathan Sokoll, Amy Todd and Rosa Castro Zarzur; Palm Associations: Gelson Tembo (Principal Investigator), Alefa Banda, Chiluba Goma, Doreen Goma, Liseteli Ndiyoi and Nathan Tembo; University of North Carolina: Sudhanshu Handa (Principal Investigator), Amber Peterman.

## Zambia MCT evaluation team

American Institutes for Research: Juan Bonilla, Alvaro Ballarin Cabrera, Thomas De Hoop, Gilbert Kiggundu, Nisha Rai, Hannah Reeves, Joshua Sennett, Dan Sherman, Jonathan Sokoll, Amy Todd and Rosa Castro Zarzur; Palm Associates: Gelson Tembo (Principal Investigator), Alefa Banda, Liseteli Ndiyoi and Nathan Tembo; University of North Carolina: Sudhanshu Handa (Principal Investigator); UNICEF Office of Research—Innocenti: Tia Palermo, Amber Peterman, Leah Prencipe.

## Zimbabwe HSCT evaluation team

American Institutes for Research: David Seidenfeld (Principal Investigator), Andi Coombes, Leah Prencipe, Hannah Reeves and Rosa Castro Zarzur; University of North Carolina: Sudhanshu Handa (Principal Investigator), Adria Molotsky, Frank Otchere and Amber Peterman; Ministry of Public Service, Labour, and Social Welfare: Sydney G. Mhishi; Center for Applied Social Sciences (CASS) Trust, University of Zimbabwe: Billy Mukamuri.

## Malawi SCT evaluation team

University of North Carolina: Sara Abdoulayi, Gustavo Angeles, Clare Barrington, Kristen Brugh, Sudhanshu Handa (Principal Investigator), Kelly Kilburn, Adria Molotsky, Frank Otchere and Susannah Zietz; Centre for Social Research, University of Malawi: Peter Mvula and Maxton Tsoka; UNICEF Office of Research—Innocenti: Jacobus de Hoop, Sudhanshu Handa, Tia Palermo and Amber Peterman.

## References

[R1] Abdoulayi S, Angeles G, Barrington C et al. 2014. Malawi social cash transfer program baseline impact evaluation report. Chapel Hill, NC: University of North Carolina at Chapel Hill.

[R2] Abdoulayi S, Angeles G, Barrington C et al. 2016. Malawi social cash transfer program endline impact evaluation report. Chapel Hill, NC: University of North Carolina at Chapel Hill.

[R3] Aiello AE, Dowd JB. 2013. Socio-economic status and immunosenescence. In: Bosch JA, Phillips AC, Lord JM (eds). *Immunosenescence*. New York: Springer, pp. 145–57.

[R4] American Institutes for Research . 2011. Zambia’s child grant programme: baseline report. Washington, DC: American Institutes for Research.

[R5] American Institutes for Research . 2014a. 12-month impact report for Zimbabwe’s harmonised social cash transfer programmes. Washington, DC: American Institutes for Research.

[R6] American Institutes for Research . 2014b. Zambia’s child grant programme: 36-month impact report Washington, DC: American Institutes for Research.

[R7] American Institutes for Research . 2015a. Zambia’s child grant program: 48 month impact report. Washington, DC: American Institutes for Research.

[R8] American Institutes for Research . 2015b. Zambia’s multiple category targeting grant: 36-month impact report. Washington, DC: American Institutes of Research.

[R9] American Institutes for Research . 2016a. Zambia’s child grant programme: 48-month impact report Washington, DC: American Institutes for Research.

[R10] American Institutes for Research . 2016b. Zambia’s multiple category targeting grant: 36-month impact report Washington, DC: American Institutes for Research.

[R11] Andrew A, Vera-Hernandez M. 2020. Incentivizing demand for supply-constrained care: Institutional birth in India. *Institute for Fiscal Studies Working Papers*. London: Institute for Fiscal Studies.

[R12] Bastagli F, Hagen-Zanker J, Harman L et al. 2019. The impact of cash transfers: a review of the evidence from low-and middle-income countries. *Journal of Social Policy* 48: 569–94.

[R13] Bonilla J, Zarzur RC, Handa S et al. 2017. Cash for women’s empowerment? A mixed-methods evaluation of the government of Zambia’s child grant program. *World Development* 95: 55–72. 31363300 10.1016/j.worlddev.2017.02.017 PMC6667169

[R14] Cai L, Kalb G. 2006. Health status and labour force participation: evidence from Australia. *Health Economics* 15: 241–61. 16229055 10.1002/hec.1053

[R15] Cai L, Mavromaras K, Oguzoglu U. 2014. The effects of health status and health shocks on hours worked. *Health Economics* 23: 516–28. 23649673 10.1002/hec.2931

[R16] Case A, Paxson C. 2008. Stature and status: height, ability, and labor market outcomes. *Journal of Political Economy* 116: 499–532. 19603086 10.1086/589524 PMC2709415

[R17] Cohen S, Janicki-Deverts D, Miller GE. 2007. Psychological stress and disease. *Jama* 298: 1685–7. 17925521 10.1001/jama.298.14.1685

[R18] Davis B, Handa S. 2015. How much do programmes pay? Transfer size in selected national cash transfer programmes in Africa. In: *Transfer Project Research Briefs*. Chapel Hill: Transfer Project, UNC Chapel Hill.

[R19] Davis B, Handa S, Hypher N et al. 2016. *From Evidence to Action: The Story of Cash Transfers and Impact Evaluation in Sub Saharan Africa*. Oxford: Oxford University Press.

[R20] De Groot R, Palermo T, Handa S, Ragno LP, Peterman A. 2017. Cash transfers and child nutrition: pathways and impacts. *Development Policy Review* 35: 621–43. 31363343 10.1111/dpr.12255 PMC6667168

[R21] De Hoop J, Groppo V, Handa S. 2020. Cash transfers, microentrepreneurial activity, and child work: evidence from Malawi and Zambia. *The World Bank Economic Review* 34: 670–97. 10.1093/wber/lhz004 PMC9997202 36909737

[R22] De Hoop J, Rosati FC. 2014. Cash transfers and child labor. *The World Bank Research Observer* 29: 202–34.

[R23] Deaton AS, Tortora R. 2015. People in sub-Saharan Africa rate their health and health care among the lowest in the world. *Health Affairs* 34: 519–27. 25715657 10.1377/hlthaff.2014.0798 PMC5674528

[R24] Dhanaraj S. 2016. Economic vulnerability to health shocks and coping strategies: evidence from Andhra Pradesh, India. *Health Policy and Planning* 31: 749–58. 26838795 10.1093/heapol/czv127

[R25] Evans DK, Holtemeyer B, Kosec K. 2017. Cash transfers and health: evidence from Tanzania. *World Bank Economic Review* 33: 394–412.

[R26] Gertler P, Heckman J, Pinto R et al. 2014. Labor market returns to an early childhood stimulation intervention in Jamaica. *Science* 344: 998–1001. 24876490 10.1126/science.1251178 PMC4574862

[R27] Ghana Leap 1000 Evaluation Team . 2016. Ghana LEAP 1000 programme: baseline evaluation report. Florence, Italy: UNICEF Office of Research.

[R28] Ghana Leap 1000 Evaluation Team . 2018. Ghana LEAP 1000 programme: endline evaluation report. Florence, Italy: UNICEF Office of Research.

[R29] Handa S, Peterman A, Huang C et al. 2015a. Impact of the Kenya cash transfer for orphans and vulnerable children on early pregnancy and marriage of adolescent girls. *Social Science & Medicine* 141: 36–45. 26246032 10.1016/j.socscimed.2015.07.024 PMC4659857

[R30] Handa S, Peterman A, Seidenfeld D, Tembo G. 2015b. Income transfers and maternal health: evidence from a national randomized social cash transfer program in Zambia. *Health Economics* 25: 225–236. 25581062 10.1002/hec.3136 PMC5488682

[R31] Helpage International . 2017. Cash transfers and older people’s access to healthcare: A multi-country study in Ethiopia, Mozambique, Tanzania and Zimbabwe. London: HelpAge International.

[R32] Hidrobo M, Hoddinott J, Kumar N, Olivier M. 2018. Social protection, food security, and asset formation. *World Development* 101: 88–103.

[R33] ILO . 2021. World social protection report 2020–22. Geneva: International Labour Office.

[R34] Lagarde M, Haines A, Palmer N. 2007. Conditional cash transfers for improving uptake of health interventions in low-and middle-income countries: a systematic review. *Jama* 298: 1900–10. 17954541 10.1001/jama.298.16.1900

[R35] Manley J, Balarajan Y, Malm S et al. 2020. Cash transfers and child nutritional outcomes: a systematic review and meta-analysis. *BMJ Global Health* 5: e003621. 10.1136/bmjgh-2020-003621 PMC7751217 33355262

[R36] Manley J, Gitter S, Slavchevska V. 2013. How effective are cash transfers at improving nutritional status? *World Development* 48: 133–55.

[R37] Mascarini-Serra L. 2011. Prevention of soil-transmitted helminth infection. *Journal of Global Infectious Diseases* 3: 175. 10.4103/0974-777X.81696 PMC3125032 21731306

[R38] Mazumdar S, Mazumdar PG, Kanjilal B, Singh PK. 2014. Multiple shocks, coping and welfare consequences: natural disasters and health shocks in the Indian Sundarbans. *PloS One* 9: e105427. 10.1371/journal.pone.0105427 PMC4149427 25170846

[R39] Mcewen BS, Seeman T. 1999. Protective and damaging effects of mediators of stress: elaborating and testing the concepts of allostasis and allostatic load. *Annals of the New York Academy of Sciences* 896: 30–47. 10681886 10.1111/j.1749-6632.1999.tb08103.x

[R40] Norton EC, Wang H, Ai C. 2004. Computing interaction effects and standard errors in logit and probit models. *The Stata Journal* 4: 154–67.

[R41] Novignon J, Nonvignon J, Arthur E. 2015. Health status and labour force participation in Sub‐Saharan Africa: a dynamic panel data analysis. *African Development Review* 27: 14–26.

[R42] Nwosu CO, Woolard I. 2017. The impact of health on labour force participation in South Africa. *South African Journal of Economics* 85: 481–90.

[R43] Onwuchekwa C, Verdonck K, Marchal B. 2021. Systematic review on the impact of conditional cash transfers on child health service utilisation and child health in sub-Saharan Africa. *Frontiers in Public Health* 9: 1–12.doi: 10.3389/fpubh.2021.643621. PMC8316722 34336755

[R44] Owusu-Addo E, Cross R. 2014. The impact of conditional cash transfers on child health in low-and middle-income countries: a systematic review. *International Journal of Public Health* 59: 609–18. 24898173 10.1007/s00038-014-0570-x

[R45] Owusu-Addo E, Renzaho AM, Smith BJ. 2018. The impact of cash transfers on social determinants of health and health inequalities in sub-Saharan Africa: a systematic review. *Health Policy and Planning* 33: 675–96. 29762708 10.1093/heapol/czy020 PMC5951115

[R46] Owusu-Addo E, Renzaho AM, Smith BJ. 2019. Cash transfers and the social determinants of health: a conceptual framework. *Health Promotion International* 34: e106–18. 30272155 10.1093/heapro/day079 PMC6913226

[R47] Pega F, Liu SY, Walter S et al. 2017. Unconditional cash transfers for reducing poverty and vulnerabilities: effect on use of health services and health outcomes in low‐and middle‐income countries. *Cochrane Database of Systematic Reviews* 2020: 1–180.doi: 10.1002/14651858.CD011135.pub2. PMC6486161 29139110

[R48] Ranganathan M, Lagarde M. 2012. Promoting healthy behaviours and improving health outcomes in low and middle income countries: a review of the impact of conditional cash transfer programmes. *Preventive Medicine* 55: S95–105. 22178043 10.1016/j.ypmed.2011.11.015

[R49] Robertson L, Mushati P, Eaton JW et al. 2013. Effects of unconditional and conditional cash transfers on child health and development in Zimbabwe: a cluster-randomised trial. *The Lancet* 381: 1283–92. 10.1016/S0140-6736(12)62168-0 PMC3627205 23453283

[R50] Roelen K, Devereux S, Abdulai A-G et al. 2017. How to make ‘cash plus’ work: linking cash transfers to services and sectors. *UNICEF Office of Research*.

[R51] Seeman TE, Crimmins E. 2001. Social environment effects on health and aging. *Annals of the New York Academy of Sciences* 954: 88–117. 11797869 10.1111/j.1749-6632.2001.tb02749.x

[R52] Seidenfeld D, Handa S, Tembo S et al. 2014. *The Impact of an Unconditional Cash Transfer on Food Security and Nutrition: The Zambia Child Grant Programme*. Brighton: Institute of Development Studies.

[R53] Steptoe A, Marmot M. 2002. The role of psychobiological pathways in socio-economic inequalities in cardiovascular disease risk. *European Heart Journal* 23: 13–25. 11741358 10.1053/euhj.2001.2611

[R54] Sun S, Huang J, Hudson DL, Sherraden M. 2020. Cash transfers and health. *Annual Review of Public Health* 42: 363–80. 10.1146/annurev-publhealth-090419-102442 33395543

[R55] Tiwari S, Daidone S, Ruvalcaba MA et al. 2016. Impact of cash transfer programs on food security and nutrition in sub-Saharan Africa: a cross-country analysis. *Global Food Security* 11: 72–83. 31396473 10.1016/j.gfs.2016.07.009 PMC6687324

[R56] Ward P, Hurrell A, Visram A et al. 2010. *Kenya CT-OVC Programme Operational and Impact Evaluation 2007–2009*. London: Oxford Policy Management.

[R57] World Bank . 2015. *The State of Social Safety Nets 2015*. Washington, DC: World Bank.

[R58] World Health Organization . 2018. The work of WHO in the African Region: report of the regional director. Geneva: WHO.

[R59] Zimmerman A, Garman E, Avendano-Pabon M et al. 2021. The impact of cash transfers on mental health in children and young people in low-income and middle-income countries: a systematic review and meta-analysis. *BMJ Global Health* 6: e004661. 10.1136/bmjgh-2020-004661 PMC8088245 33906845

