# Supplementary material for: The impact of unconditional cash transfers on morbidity and health-seeking behaviour in Africa: evidence from Ghana, Malawi, Zambia and Zimbabwe
Source: Health Policy Plan. 2022 Feb 14;37(5):607–23. doi: 10.1093/heapol/czac014 (PMC9113146; doi:10.1093/heapol/czac014)
Supplement: czac014_Supp [file czac014_supp.zip › Appendices - v3.docx]

**Appendix 1. Health Outcomes Descriptions by Country**

| Variable | Ghana | Malawi | Zambia CGP/MCT | Zimbabwe |
| --- | --- | --- | --- | --- |
| Incidence of illness (fever/malaria, respiratory and diarrhea) | Has [NAME] been sick or injured during the **last two weeks**? | During the past 2 weeks has [name] suffered from an illness or injury? What was the most recent illness or injury? | Has [name] been sick or injured during the last two weeks? What was [name] mainly suffering from? | Was [name] sick or injured during the last thirty days? What was [name] mainly suffering from? |
| Seeking medical care (i.e., health seeking) | During the last 2 weeks has [NAME] consulted a health practitioner, visited a health facility or consulted a traditional healer for this injury/illness? | What action did [name] take to find relief for the illness or injury? Response options include nothing, used medicine had in stock, sought treatment at a public facility, sought treatment at a private/church/mission facility; went to local pharmacy; sought treatment with traditional healer; other | Did [name] consult any health or other institution/persons for this illness/injury or did he/she only use self-administered medicine? Response options include: consulted, self-administered medicine only, none of the above. | What was the main health facility visited? Response options include: public health facility, private health facility, mission health facility, mine/farm health facility, traditional healer, pharmacy, other, none. |
| Health spending (total expenditure in local currency unit) | How much in total was spent on [NAME]’s medication and consultation **in the last two weeks?**  How much in total was spent on [NAME]’s medicine or medical supplies **in the last two weeks?** | 1) How much in total did household spend in the past 4 weeks for all of [name]’s illnesses and injuries, including for medicine, tests, consultation and in-patient fees, if any? 2) How much in total did household spend on [name] in the past 4 weeks for medical care not related to an illness: preventative health care, prenatal visits, check-ups, etc.? | How much in total was spent on [name] medication and consultation in the last two weeks? Including both cash and in kind | How much in total was spend on medical consultations, treatment, and drugs for [name] in the last thirty days? Including both cash and in kind. |
| Self-assessed health | n/a | How would you rate [name]’s health in general? Responses include poor, fair, good, very good, excellent. Variable coded 1-5 with 1 being poor and 5 being excellent. | How would you rate [name]’s health in general? Responses include poor, fair, good, very good, excellent. Variable coded 1-5 with 1 being excellent and 5 being poor. Recoded for analysis. | How would you rate [name]’s health in general? Responses include poor, fair, good, very good, excellent. Variable coded 1-5 with 1 being poor and 5 being excellent. |

**APPENDIX 2. Indicator construction and availability, by country**

| Indicator | Variable type | Variable construction | Ghana | Malawi | Zambia CGP | Zambia MCT | Zimbabwe |
| --- | --- | --- | --- | --- | --- | --- | --- |
| **CONTROLS** |  |  |  |  |  |  |  |
| Age in years | Continuous | Age of individual (in years for individuals ages 5 and above; in months for children under 5 years; in Kenya measured as completed years (e.g., ‘0’ if child under 1 year)) | ✔ | ✔ | ✔ | ✔ | ✔ |
| Sex | Binary | Coded as 1 if respondent is male and 0, if female | ✔ | ✔ | ✔ | ✔ | ✔ |
| Attended school | Binary | Coded as 1 if respondent has ever been to school |  | ✔ | ✔ | ✔ |  |
| Household size | Continuous | Total number of people in the household | ✔ | ✔ | ✔ | ✔ | ✔ |
| Household has access to clean water source | Binary | Binary for access to clean water in household (1=yes) | ✔ | ✔ | ✔ | ✔ | ✔ |
| Household has access to improved toilet facilities | Binary | Binary for access to some toilet facility in household (1=yes) | ✔ | ✔ | ✔ | ✔ |  |
| Household was affected by any shock | Binary | Binary for household affected by any shock (1=yes) |  | ✔ | ✔ | ✔ |  |
| Per capita monthly expenditure | Continuous | Total household monthly per capita expenditure in local currency unit | ✔ | ✔ | ✔ | ✔ |  |
| District fixed effects | Binary | Dummary variable =1 for each district (excluding reference) | ✔ (community) | ✔ | ✔ | ✔ | ✔ |
| **OUTCOMES** |  |  |  |  |  |  |  |
| Health expenditure | Continuous | See Appendix 1 | ✔ | ✔ | ✔ | ✔ | ✔ |
| Self-assessed health | Interval: range 1-5 | See Appendix 1 |  | ✔ | ✔ | ✔ | ✔ |
| Illness | Binary | See Appendix 1 | ✔ | ✔ | ✔ | ✔ | ✔ |
| Sought medical care | Binary | See Appendix 1 | ✔ | ✔ | ✔ | ✔ | ✔ |
| Chronic illness | Binary | See Appendix 1 |  | ✔ | ✔ | ✔ | ✔ |
| Malaria | Binary | See Appendix 1 |  | ✔ | ✔ | ✔ | ✔ |
| Respiratory | Binary | See Appendix 1 | ✔ | ✔ | ✔ | ✔ | ✔ |
| Diarrhea | Binary | See Appendix 1 | ✔ | ✔ | ✔ | ✔ | ✔ |
| Diarrhea - sought care | Binary | See Appendix 1 | ✔ | ✔ | ✔ | ✔ | ✔ |
| Fever | Binary | See Appendix 1 | ✔ | ✔ | ✔ | ✔ | ✔ |
| Fever - sought care | Binary | See Appendix 1 | ✔ | ✔ | ✔ | ✔ | ✔ |
| Cough | Binary | See Appendix 1 | ✔ | ✔ | ✔ | ✔ | ✔ |
| Cough – sought care | Binary | See Appendix 1 | ✔ | ✔ | ✔ | ✔ | ✔ |

Notes: Head refers to head of household, and respondent refers to main respondent answering the survey on behalf of all household members. In Ghana, shocks were only collected at endline so it was not included as a covariate in models.

**Appendix 3. Differential attrition analysis across countries and survey waves**

|  | Full sample | Children 0 - 5 years | Children 5 - 19 years | Adults 20 - 59 years | Adults 60+ years |
| --- | --- | --- | --- | --- | --- |
| **Panel A: Ghana LEAP 1000** | | | | | |
| Treatment indicator | -0.009 | -0.006 | -0.008 | -0.002 | -0.037 |
|  | (0.70) | (0.39) | (0.35) | (0.15) | (0.66) |
| Constant | 1.467** | 1.571** | 1.542 | 0.989 | 4.398** |
|  | (2.30) | (2.42) | (1.37) | (1.25) | (2.01) |
| *N* | 15,512 | 3,911 | 5,346 | 5,461 | 794 |

**Panel B: Malawi SCTP at 24 months**

| Treatment indicator | 0.000 | 0.000 | -0.000 | -0.002 | -0.009 |
| --- | --- | --- | --- | --- | --- |
|  | (0.01) |  | (0.69) | (0.39) | (0.30) |
| Constant | 0.025*** | 0.000 | 0.001* | 0.013*** | 0.136*** |
|  | (4.93) |  | (1.72) | (2.88) | (6.33) |
| *N* | 15,251 | 1,848 | 8,026 | 2,933 | 2,444 |

**Panel C1: Zambia CGP at 24 months**

| Treatment indicator | 0.006 | 0.020 | 0.003 | -0.006 | 0.100 |
| --- | --- | --- | --- | --- | --- |
|  | (0.23) | (0.73) | (0.10) | (0.26) | (1.12) |
| Constant | 0.830*** | 0.843*** | 0.810*** | 0.850*** | 0.640*** |
|  | (47.78) | (41.33) | (42.16) | (52.88) | (9.25) |
| *N* | 14,156 | 4,121 | 5,302 | 4,549 | 184 |

**Panel C2: Zambia CGP at 36 months**

| Treatment indicator | -0.007 | -0.016 | -0.004 | -0.001 | -0.033 |
| --- | --- | --- | --- | --- | --- |
|  | (0.26) | (0.60) | (0.17) | (0.04) | (0.67) |
| Constant | 0.112*** | 0.102*** | 0.135*** | 0.094*** | 0.148*** |
|  | (6.39) | (5.43) | (8.33) | (4.50) | (3.90) |
| *N* | 14,156 | 4,121 | 5,302 | 4,549 | 184 |

**Panel C3: Zambia CGP at 48 months**

| Treatment indicator | -0.012 | -0.013 | -0.018 | -0.004 | -0.014 |
| --- | --- | --- | --- | --- | --- |
|  | (0.48) | (0.50) | (0.75) | (0.16) | (0.25) |
| Constant | 0.162*** | 0.135*** | 0.205*** | 0.136*** | 0.182*** |
|  | (9.42) | (6.93) | (12.52) | (7.05) | (4.50) |
| *N* | 14,156 | 4,121 | 5,302 | 4,549 | 184 |

**Panel D1: Zambia MCT at 24 months**

| Treatment indicator | -0.030* | -0.054 | -0.030** | -0.022 | -0.023 |
| --- | --- | --- | --- | --- | --- |
|  | (1.86) | (1.62) | (2.00) | (1.01) | (1.31) |
| Constant | 0.860*** | 0.847*** | 0.869*** | 0.824*** | 0.905*** |
|  | (78.78) | (45.23) | (84.66) | (52.20) | (83.83) |
| *N* | 14,959 | 1,662 | 7,334 | 3,910 | 2,053 |

**Panel D2: Zambia MCT at 36 months**

| Treatment indicator | -0.032** | -0.056* | -0.029 | -0.032 | -0.026 |
| --- | --- | --- | --- | --- | --- |
|  | (2.08) | (1.92) | (1.66) | (1.59) | (1.48) |
| Constant | 0.832*** | 0.844*** | 0.838*** | 0.801*** | 0.858*** |
|  | (85.42) | (45.56) | (82.58) | (55.84) | (76.95) |
| *N* | 14,959 | 1,662 | 7,334 | 3,910 | 2,053 |

**Panel E: Zimbabwe 12 months**

| Treatment indicator | 0.034 | 0.023 | 0.042 | 0.028 | 0.022 |
| --- | --- | --- | --- | --- | --- |
|  | (1.23) | (0.63) | (1.40) | (0.90) | (0.75) |
| Constant | 0.728*** | 0.707*** | 0.725*** | 0.693*** | 0.801*** |
|  | (33.40) | (25.31) | (29.53) | (31.32) | (31.59) |
| *N* | 14,496 | 1,748 | 7,020 | 3,060 | 2,668 |

Notes: The above estimates are obtained using cross-sectional OLS regressions of the main indicator variables by programme and follow-up wave on the treatment dummy and a constant. In Ghana, the PMT score is added as an additional covariate, but results are robust to exclusion of the PMT score. Robust t-statistics in parentheses. * *p*<0.1; ** *p*<0.05; *** *p*<0.01.

**Appendix 4. Baseline means of background characteristics and outcomes at baseline, by treatment status and age group**

**GHANA**

|  | Pooled | | | | Comparison | | | | Treatment | | | | |  |
| --- | --- | --- | --- | --- | --- | --- | --- | --- | --- | --- | --- | --- | --- | --- |
|  | Mean | | N | | Mean | | N | | Mean | | N | | | p-value |
| **Panel A: Ages 0 – 4** | | | | | | | | | | | | | | |
| Age | 1.604 | | 3,911 | | 1.609 | | 1,873 | | 1.599 | | 2,038 | | | 0.575 |
| Female | 0.500 | | 3,911 | | 0.500 | | 1,873 | | 0.500 | | 2,038 | | | 0.024 |
| Head no formal schooling | 0.815 | | 3,911 | | 0.793 | | 1,873 | | 0.835 | | 2,038 | | | 0.908 |
| Improved source of water | 0.569 | | 3,911 | | 0.552 | | 1,873 | | 0.585 | | 2,038 | | | 0.797 |
| Improved source of sanitation | 0.102 | | 3,911 | | 0.097 | | 1,873 | | 0.106 | | 2,038 | | | 0.507 |
| Household size | 7.268 | | 3,911 | | 6.912 | | 1,873 | | 7.595 | | 2,038 | | | 0.263 |
| Total household monthly per capita expenditure (Ghana cedis) | 62.393 | | 3,911 | | 65.037 | | 1,873 | | 59.964 | | 2,038 | | | 0.870 |
| Preventive care | 0.424 | | 3,521 | | 0.421 | | 1,697 | | 0.428 | | 1,824 | | | 0.608 |
| Diarrhoea last 2 weeks | 0.391 | | 3,521 | | 0.410 | | 1,697 | | 0.372 | | 1,824 | | | 0.018 |
| Sought care for diarrhoea | 0.906 | | 1,375 | | 0.908 | | 696 | | 0.904 | | 679 | | | 0.670 |
| Fever last 2 weeks | 0.249 | | 3,521 | | 0.269 | | 1,697 | | 0.231 | | 1,824 | | | 0.006 |
| Sought care for fever | 0.994 | | 878 | | 0.996 | | 456 | | 0.993 | | 422 | | | 0.803 |
| Symptoms of ARI last 2 weeks | 0.055 | | 3,521 | | 0.058 | | 1,697 | | 0.052 | | 1,824 | | | 0.743 |
| Sought care for ari | 1.000 | | 193 | | 1.000 | | 99 | | 1.000 | | 94 | | |  |
| Illness in last two weeks (child < 5) | 0.572 | | 3,521 | | 0.593 | | 1,697 | | 0.552 | | 1,824 | | | 0.074 |
| Sought care for illness (child < 5) | 0.504 | | 2,013 | | 0.518 | | 1,007 | | 0.490 | | 1,006 | | | 0.778 |
| Real child health expenditures | 16.041 | | 3,521 | | 14.249 | | 1,697 | | 17.709 | | 1,824 | | | 0.298 |
| **Panel b: Ages 5 – 19** | | | | | | | | | | | | | | |
| Age | 9.972 | | 5,346 | | 10.023 | | 2,338 | | 9.933 | | 3,008 | | | 0.653 |
| Female | 0.482 | | 5,346 | | 0.487 | | 2,338 | | 0.479 | | 3,008 | | | 0.869 |
| Head no formal schooling | 0.848 | | 5,346 | | 0.833 | | 2,338 | | 0.859 | | 3,008 | | | 0.419 |
| Improved source of water | 0.577 | | 5,346 | | 0.565 | | 2,338 | | 0.586 | | 3,008 | | | 0.429 |
| Improved source of sanitation | 0.102 | | 5,346 | | 0.095 | | 2,338 | | 0.108 | | 3,008 | | | 0.860 |
| Household size | 8.480 | | 5,346 | | 8.187 | | 2,338 | | 8.707 | | 3,008 | | | 0.428 |
| Total household monthly per capita expenditure (Ghana cedis) | 57.779 | | 5,346 | | 58.404 | | 2,338 | | 57.294 | | 3,008 | | | 0.146 |
| Illness in last 2 weeks | 0.206 | | 5,334 | | 0.204 | | 2,332 | | 0.207 | | 3,002 | | | 0.543 |
| Sought care for illness in last 2 weeks | 0.535 | | 1,098 | | 0.527 | | 476 | | 0.540 | | 622 | | | 0.628 |
| Real health expenditures | 4.493 | | 5,346 | | 4.683 | | 2,338 | | 4.345 | | 3,008 | | | 0.928 |
| **Panel C: Ages 20 – 59** | | | | | | | | | | | | | | |
| Age | 33.832 | | 5,461 | | 33.321 | | 2,688 | | 34.328 | | 2,773 | | | 0.808 |
| Female | 0.568 | | 5,461 | | 0.565 | | 2,688 | | 0.572 | | 2,773 | | | 0.351 |
| Head no formal schooling | 0.798 | | 5,461 | | 0.777 | | 2,688 | | 0.818 | | 2,773 | | | 0.561 |
| Improved source of water | 0.586 | | 5,461 | | 0.574 | | 2,688 | | 0.598 | | 2,773 | | | 0.785 |
| Improved source of sanitation | 0.099 | | 5,461 | | 0.100 | | 2,688 | | 0.098 | | 2,773 | | | 0.803 |
| Household size | 7.247 | | 5,461 | | 6.892 | | 2,688 | | 7.591 | | 2,773 | | | 0.190 |
| Total household monthly per capita expenditure (Ghana cedis) | 62.787 | | 5,461 | | 64.471 | | 2,688 | | 61.155 | | 2,773 | | | 0.168 |
| Illness in last 2 weeks | 0.255 | | 5,443 | | 0.258 | | 2,678 | | 0.252 | | 2,765 | | | 0.291 |
| Sought care for illness in last 2 weeks | 0.565 | | 1,387 | | 0.575 | | 691 | | 0.555 | | 696 | | | 0.088 |
| Real health expenditures | 8.477 | | 5,461 | | 8.294 | | 2,688 | | 8.654 | | 2,773 | | | 0.629 |
| **Panel D: Ages 60 plus** | | | | | | | | | | | | | | |
| Age | | 70.499 | | 794 | | 70.363 | | 375 | | 70.621 | | 419 | 0.800 | |
| Female | | 0.560 | | 794 | | 0.573 | | 375 | | 0.549 | | 419 | 0.247 | |
| Head no formal schooling | | 0.788 | | 794 | | 0.784 | | 375 | | 0.792 | | 419 | 0.829 | |
| Improved source of water | | 0.612 | | 794 | | 0.611 | | 375 | | 0.613 | | 419 | 0.761 | |
| Improved source of sanitation | | 0.079 | | 794 | | 0.059 | | 375 | | 0.098 | | 419 | 0.509 | |
| Household size | | 7.461 | | 794 | | 7.232 | | 375 | | 7.666 | | 419 | 0.493 | |
| Total household monthly per capita expenditure (Ghana cedis) | | 57.855 | | 794 | | 61.555 | | 375 | | 54.544 | | 419 | 0.746 | |
| Illness in last 2 weeks | | 0.215 | | 794 | | 0.213 | | 375 | | 0.217 | | 419 | 0.448 | |
| Sought care for illness in last 2 weeks | | 0.468 | | 171 | | 0.450 | | 80 | | 0.484 | | 91 | 0.763 | |
| Real health expenditures | | 7.042 | | 794 | | 7.034 | | 375 | | 7.050 | | 419 | 0.308 | |

Notes: Bivariate regressions test difference between treatment and control groups, controlling for PMT Score. Standard errors are clustered at the community level.

**MALAWI**

|  | | Pooled | | | | Control | | | | Treatment | | | |  | |
| --- | --- | --- | --- | --- | --- | --- | --- | --- | --- | --- | --- | --- | --- | --- | --- |
| Variables | | Mean | | N | | Mean | | N | | Mean | | N | | p-value | |
| **Panel A: Children under 5** | | | | | | | | | | | | | | | |
| Age in years | | 2.22 | | 1,848 | | 2.24 | | 976 | | 2.20 | | 872 | | 0.66 | |
| Male | | 1.51 | | 1,848 | | 1.51 | | 976 | | 1.51 | | 872 | | 0.70 | |
| Recipient attended school | | 0.41 | | 1,848 | | 0.42 | | 976 | | 0.40 | | 872 | | 0.93 | |
| Household has access to some toilet facilities | | 0.75 | | 1,848 | | 0.72 | | 976 | | 0.77 | | 872 | | 0.35 | |
| Household has access to clean water source | | 0.89 | | 1,848 | | 0.88 | | 976 | | 0.89 | | 872 | | 0.88 | |
| Household was affected by any shock | | 0.96 | | 1,848 | | 0.95 | | 976 | | 0.97 | | 872 | | 0.64 | |
| Household size | | 6.46 | | 1,848 | | 6.44 | | 976 | | 6.48 | | 872 | | 0.88 | |
| Total household monthly per capita expenditure (Malawian Kwacha) | | 2,581.12 | | 1,848 | | 2,521.27 | | 976 | | 2,643.33 | | 872 | | 0.83 | |
| Salima district | | 0.44 | | 1,848 | | 0.48 | | 976 | | 0.39 | | 872 | | 0.72 | |
| Preventive care | | 0.50 | | 1,746 | | 0.52 | | 916 | | 0.48 | | 830 | | 0.79 | |
| Diarrhoea past 2 weeks | | 0.16 | | 1,746 | | 0.16 | | 916 | | 0.17 | | 830 | | 0.79 | |
| Diarrhoea care | | 0.62 | | 298 | | 0.68 | | 154 | | 0.57 | | 144 | | 0.41 | |
| Fever past 2 weeks | | 0.26 | | 1,746 | | 0.28 | | 916 | | 0.24 | | 830 | | 0.47 | |
| Fever care | | 0.61 | | 478 | | 0.67 | | 264 | | 0.55 | | 214 | | 0.21 | |
| Cough past 2 weeks | | 0.26 | | 1,746 | | 0.26 | | 916 | | 0.26 | | 830 | | 0.77 | |
| Cough care | | 0.60 | | 466 | | 0.62 | | 249 | | 0.57 | | 217 | | 0.63 | |
| Health expenditure (Malawian Kwacha) | | 45.51 | | 1,845 | | 40.46 | | 975 | | 50.76 | | 870 | | 0.45 | |
| **Panel B: Ages 5-19** | | | | | | | | | | | | | | | |
| Age in years | | 11.00 | | 8,026 | | 10.92 | | 4,249 | | 11.09 | | 3,777 | | 0.27 | |
| Male | | 1.49 | | 8,026 | | 1.49 | | 4,249 | | 1.49 | | 3,777 | | 0.80 | |
| Recipient attended school | | 0.34 | | 8,026 | | 0.34 | | 4,249 | | 0.33 | | 3,777 | | 0.79 | |
| Household has access to some toilet facilities | | 0.76 | | 8,026 | | 0.74 | | 4,249 | | 0.78 | | 3,777 | | 0.41 | |
| Household has access to clean water source | | 0.90 | | 8,026 | | 0.90 | | 4,249 | | 0.90 | | 3,777 | | 0.98 | |
| Household was affected by any shock | | 0.96 | | 8,026 | | 0.94 | | 4,249 | | 0.97 | | 3,777 | | 0.40 | |
| Household size | | 5.90 | | 8,026 | | 5.90 | | 4,249 | | 5.89 | | 3,777 | | 0.87 | |
| Total household monthly per capita expenditure (Malawian Kwacha) | | 2,871.08 | | 8,026 | | 2,823.01 | | 4,249 | | 2,921.74 | | 3,777 | | 0.86 | |
| Salima district | | 0.42 | | 8,026 | | 0.45 | | 4,249 | | 0.39 | | 3,777 | | 0.78 | |
| Illness | | 0.17 | | 8,026 | | 0.16 | | 4,249 | | 0.18 | | 3,777 | | 0.22 | |
| Seek medical care | | 0.54 | | 1,420 | | 0.61 | | 703 | | 0.48 | | 717 | | **0.00** | |
| Chronic illness | | 0.07 | | 4,954 | | 0.06 | | 2,591 | | 0.08 | | 2,363 | | 0.40 | |
| Malaria | | 0.05 | | 8,026 | | 0.05 | | 4,249 | | 0.05 | | 3,777 | | 0.44 | |
| Respiratory | | 0.06 | | 8,026 | | 0.06 | | 4,249 | | 0.06 | | 3,777 | | 1.00 | |
| Diarrhoea | | 0.02 | | 8,026 | | 0.02 | | 4,249 | | 0.02 | | 3,777 | | 0.82 | |
| Health expenditure (Malawian Kwacha) | | 34.91 | | 8,016 | | 37.14 | | 4,243 | | 32.57 | | 3,773 | | 0.81 | |
| Self-assessed health | | 0.51 | | 8,000 | | 0.47 | | 4,235 | | 0.55 | | 3,765 | | 0.39 | |
| **Panel C: Ages 20-59** | | | | | | | | | | | | | | | |
| Age in years | | 37.22 | | 2,933 | | 37.57 | | 1,562 | | 36.84 | | 1,371 | | 0.32 | |
| Male | | 1.69 | | 2,933 | | 1.70 | | 1,562 | | 1.68 | | 1,371 | | 0.41 | |
| Recipient attended school | | 0.39 | | 2,933 | | 0.40 | | 1,562 | | 0.38 | | 1,371 | | 0.99 | |
| Household has access to some toilet facilities | | 0.80 | | 2,933 | | 0.79 | | 1,562 | | 0.82 | | 1,371 | | 0.50 | |
| Household has access to clean water source | | 0.90 | | 2,933 | | 0.89 | | 1,562 | | 0.90 | | 1,371 | | 0.97 | |
| Household was affected by any shock | | 0.96 | | 2,933 | | 0.95 | | 1,562 | | 0.97 | | 1,371 | | 0.46 | |
| Household size | | 5.91 | | 2,933 | | 5.84 | | 1,562 | | 6.00 | | 1,371 | | 0.54 | |
| Total household monthly per capita expenditure (Malawian Kwacha) | | 3,125.96 | | 2,933 | | 3,142.36 | | 1,562 | | 3,108.46 | | 1,371 | | 0.71 | |
| Salima district | | 0.45 | | 2,933 | | 0.47 | | 1,562 | | 0.43 | | 1,371 | | 0.90 | |
| Illness | | 0.30 | | 2,933 | | 0.29 | | 1,562 | | 0.30 | | 1,371 | | 0.57 | |
| Seek medical care | | 0.60 | | 896 | | 0.62 | | 461 | | 0.57 | | 435 | | 0.08 | |
| Chronic illness | | 0.22 | | 2,933 | | 0.21 | | 1,562 | | 0.24 | | 1,371 | | 0.72 | |
| Malaria | | 0.09 | | 2,933 | | 0.08 | | 1,562 | | 0.10 | | 1,371 | | 0.33 | |
| Respiratory | | 0.08 | | 2,933 | | 0.09 | | 1,562 | | 0.07 | | 1,371 | | 0.20 | |
| Diarrhoea | | 0.02 | | 2,933 | | 0.02 | | 1,562 | | 0.03 | | 1,371 | | 0.52 | |
| Health expenditure (Malawian Kwacha) | | 101.92 | | 2,929 | | 97.03 | | 1,558 | | 107.12 | | 1,371 | | 0.85 | |
| Self-assessed health | | 0.37 | | 2,925 | | 0.34 | | 1,560 | | 0.41 | | 1,365 | | 0.47 | |
| **Panel D: Ages 60 plus** | | | | | | | | | | | | | | | |
| Age in years | | 74.19 | | 2,444 | | 74.04 | | 1,230 | | 74.32 | | 1,214 | | 0.66 | |
| Male | | 1.74 | | 2,444 | | 1.74 | | 1,230 | | 1.74 | | 1,214 | | 0.56 | |
| Recipient attended school | | 0.22 | | 2,444 | | 0.21 | | 1,230 | | 0.23 | | 1,214 | | 0.56 | |
| Household has access to some toilet facilities | | 0.76 | | 2,444 | | 0.75 | | 1,230 | | 0.77 | | 1,214 | | 0.71 | |
| Household has access to clean water source | | 0.89 | | 2,444 | | 0.91 | | 1,230 | | 0.87 | | 1,214 | | 0.29 | |
| Household was affected by any shock | | 0.93 | | 2,444 | | 0.92 | | 1,230 | | 0.93 | | 1,214 | | 1.00 | |
| Household size | | 3.96 | | 2,444 | | 4.02 | | 1,230 | | 3.91 | | 1,214 | | 0.65 | |
| Total household monthly per capita expenditure (Malawian Kwacha) | | 4,227.75 | | 2,444 | | 4,064.77 | | 1,230 | | 4,379.29 | | 1,214 | | 0.39 | |
| Salima district | | 0.35 | | 2,444 | | 0.39 | | 1,230 | | 0.31 | | 1,214 | | 0.74 | |
| Illness | | 0.56 | | 2,444 | | 0.53 | | 1,230 | | 0.59 | | 1,214 | | **0.01** | |
| Seek medical care | | 0.48 | | 1,357 | | 0.50 | | 640 | | 0.46 | | 717 | | 0.35 | |
| Chronic illness | | 0.59 | | 2,441 | | 0.57 | | 1,229 | | 0.60 | | 1,212 | | 0.62 | |
| Malaria | | 0.11 | | 2,444 | | 0.10 | | 1,230 | | 0.11 | | 1,214 | | 0.41 | |
| Respiratory | | 0.19 | | 2,444 | | 0.19 | | 1,230 | | 0.20 | | 1,214 | | 0.35 | |
| Diarrhoea | | 0.04 | | 2,444 | | 0.04 | | 1,230 | | 0.04 | | 1,214 | | 0.92 | |
| Health expenditure (Malawian Kwacha) | | 209.42 | | 2,442 | | 177.38 | | 1,229 | | 239.22 | | 1,213 | | 0.27 | |
| Self-assessed health | | 0.14 | | 2,432 | | 0.13 | | 1,223 | | 0.14 | | 1,209 | | 0.96 | |

Notes: Bivariate regressions test difference between treatment and control groups. Self-assessed health only available for respondents 18 and older. Standard errors are clustered at the community level. Bold denotes significance at the alpha = 0.05 level.

**ZAMBIA CGP**

|  | | Pooled | | | | Control | | | | Treatment | | | | | |  | | |
| --- | --- | --- | --- | --- | --- | --- | --- | --- | --- | --- | --- | --- | --- | --- | --- | --- | --- | --- |
| Variables | | Mean | | N | | Mean | | N | | Mean | | | N | | | p-value | | |
| **Panel A: Children under 5** | | | | | | | | | | | | | | | | | | |
| Age in years | | 1.79 | | 4,121 | | 1.81 | | 2,066 | | 1.77 | | | 2,055 | | | 0.33 | | |
| Male | | 0.49 | | 4,121 | | 0.50 | | 2,066 | | 0.48 | | | 2,055 | | | 0.16 | | |
| Recipient attended school | | 0.72 | | 4,121 | | 0.71 | | 2,066 | | 0.74 | | | 2,055 | | | 0.43 | | |
| Household has access to some toilet facilities | | 0.51 | | 4,121 | | 0.52 | | 2,066 | | 0.51 | | | 2,055 | | | 0.90 | | |
| Household has access to clean water source | | 0.22 | | 4,121 | | 0.21 | | 2,066 | | 0.23 | | | 2,055 | | | 0.79 | | |
| Household was affected by any shock | | 0.21 | | 4,121 | | 0.22 | | 2,066 | | 0.19 | | | 2,055 | | | 0.71 | | |
| Household size | | 6.00 | | 4,121 | | 5.90 | | 2,066 | | 6.09 | | | 2,055 | | | 0.32 | | |
| Total household monthly per capita expenditure(Zambian Kwacha) | | 37.99 | | 4,121 | | 37.13 | | 2,066 | | 38.85 | | | 2,055 | | | 0.50 | | |
| kaputa district | | 0.37 | | 4,121 | | 0.38 | | 2,066 | | 0.36 | | | 2,055 | | | 0.89 | | |
| shangombo district | | 0.32 | | 4,121 | | 0.32 | | 2,066 | | 0.33 | | | 2,055 | | | 0.92 | | |
| child preventive care | | 0.78 | | 4,071 | | 0.76 | | 2,043 | | 0.80 | | | 2,028 | | | 0.15 | | |
| child diarrhoea | | 0.19 | | 4,057 | | 0.18 | | 2,038 | | 0.20 | | | 2,019 | | | 0.29 | | |
| Treated child diarrhoea | | 0.76 | | 778 | | 0.75 | | 367 | | 0.76 | | | 411 | | | 0.81 | | |
| child fever | | 0.23 | | 4,082 | | 0.23 | | 2,048 | | 0.24 | | | 2,034 | | | 0.82 | | |
| Treated child fever | | 0.74 | | 954 | | 0.74 | | 472 | | 0.74 | | | 482 | | | 0.92 | | |
| child cough | | 0.23 | | 4,082 | | 0.23 | | 2,048 | | 0.24 | | | 2,034 | | | 0.82 | | |
| Treated child cough | | 0.20 | | 4,081 | | 0.21 | | 2,047 | | 0.20 | | | 2,034 | | | 0.84 | | |
| Health expenditure (Zambian Kwacha) | | 1,109.41 | | 862 | | 1,028.28 | | 428 | | 1,189.42 | | | 434 | | | 0.67 | | |
| **Panel B: Ages 5-19** | | | | | | | | | | | | | | | | | | |
| Age in years | | 10.38 | | 5,302 | | 10.22 | | 2,602 | | 10.54 | | | 2,700 | | | **0.01** | | |
| Male | | 0.49 | | 5,302 | | 0.50 | | 2,602 | | 0.48 | | | 2,700 | | | 0.07 | | |
| Recipient attended school | | 0.72 | | 5,302 | | 0.71 | | 2,602 | | 0.74 | | | 2,700 | | | 0.58 | | |
| Household has access to some toilet facilities | | 0.54 | | 5,302 | | 0.53 | | 2,602 | | 0.54 | | | 2,700 | | | 0.92 | | |
| Household has access to clean water source | | 0.23 | | 5,302 | | 0.24 | | 2,602 | | 0.23 | | | 2,700 | | | 0.93 | | |
| Household was affected by any shock | | 0.20 | | 5,302 | | 0.20 | | 2,602 | | 0.21 | | | 2,700 | | | 0.89 | | |
| Household size | | 7.13 | | 5,302 | | 7.00 | | 2,602 | | 7.26 | | | 2,700 | | | 0.21 | | |
| Total household monthly per capita expenditure(Zambian Kwacha) | | 34.58 | | 5,302 | | 33.45 | | 2,602 | | 35.67 | | | 2,700 | | | 0.33 | | |
| kaputa district | | 0.36 | | 5,302 | | 0.35 | | 2,602 | | 0.37 | | | 2,700 | | | 0.86 | | |
| shangombo district | | 0.33 | | 5,302 | | 0.34 | | 2,602 | | 0.32 | | | 2,700 | | | 0.86 | | |
| Illness | | 0.10 | | 5,302 | | 0.10 | | 2,602 | | 0.10 | | | 2,700 | | | 0.89 | | |
| Seek medical care | | 0.69 | | 529 | | 0.67 | | 261 | | 0.72 | | | 268 | | | 0.31 | | |
| Chronic illness | | 0.01 | | 5,296 | | 0.01 | | 2,599 | | 0.01 | | | 2,697 | | | 0.70 | | |
| Malaria | | 0.03 | | 5,302 | | 0.03 | | 2,602 | | 0.02 | | | 2,700 | | | 0.58 | | |
| Respiratory | | 0.02 | | 5,302 | | 0.03 | | 2,602 | | 0.02 | | | 2,700 | | | 0.15 | | |
| Diarrhoea | | 0.01 | | 5,302 | | 0.01 | | 2,602 | | 0.02 | | | 2,700 | | | 0.13 | | |
| Health expenditure (Zambian Kwacha) | | 6,938.55 | | 436 | | 1,345.13 | | 215 | | 12,380.11 | | | 221 | | | 0.15 | | |
| **Panel C: Ages 20-59** | | | | | | | | | | | | | | | | | | |
| Age in years | | 31.91 | | 4,549 | | 31.71 | | 2,249 | | 32.10 | | 2,300 | | | 0.32 | | |  |
| Male | | 0.44 | | 4,549 | | 0.44 | | 2,249 | | 0.45 | | 2,300 | | | 0.39 | | |  |
| Recipient attended school | | 0.73 | | 4,549 | | 0.71 | | 2,249 | | 0.75 | | 2,300 | | | 0.38 | | |  |
| Household has access to some toilet facilities | | 0.51 | | 4,549 | | 0.51 | | 2,249 | | 0.51 | | 2,300 | | | 0.99 | | |  |
| Household has access to clean water source | | 0.23 | | 4,549 | | 0.23 | | 2,249 | | 0.23 | | 2,300 | | | 0.89 | | |  |
| Household was affected by any shock | | 0.20 | | 4,549 | | 0.20 | | 2,249 | | 0.19 | | 2,300 | | | 0.88 | | |  |
| Household size | | 6.15 | | 4,549 | | 6.04 | | 2,249 | | 6.25 | | 2,300 | | | 0.34 | | |  |
| Total household monthly per capita expenditure(Zambian Kwacha) | | 39.55 | | 4,549 | | 38.37 | | 2,249 | | 40.71 | | 2,300 | | | 0.36 | | |  |
| kaputa district | | 0.35 | | 4,549 | | 0.35 | | 2,249 | | 0.35 | | 2,300 | | | 0.97 | | |  |
| shangombo district | | 0.34 | | 4,549 | | 0.34 | | 2,249 | | 0.34 | | 2,300 | | | 0.98 | | |  |
| Illness | | 0.15 | | 4,549 | | 0.15 | | 2,249 | | 0.15 | | 2,300 | | | 0.88 | | |  |
| Seek medical care | | 0.64 | | 670 | | 0.65 | | 336 | | 0.63 | | 334 | | | 0.69 | | |  |
| Chronic illness | | 0.03 | | 4,547 | | 0.03 | | 2,248 | | 0.03 | | 2,299 | | | 0.93 | | |  |
| Malaria | | 0.02 | | 4,549 | | 0.02 | | 2,249 | | 0.02 | | 2,300 | | | 0.83 | | |  |
| Respiratory | | 0.03 | | 4,549 | | 0.03 | | 2,249 | | 0.02 | | 2,300 | | | 0.15 | | |  |
| Diarrhoea | | 0.03 | | 4,549 | | 0.02 | | 2,249 | | 0.03 | | 2,300 | | | 0.55 | | |  |
| Health expenditure (Zambian Kwacha) | | 3,669.06 | | 513 | | 3,948.08 | | 258 | | 3,386.75 | | 255 | | | 0.84 | | |  |
| Self-assessed health | | 0.47 | | 4,537 | | 0.48 | | 2,246 | | 0.46 | | 2,291 | | | 0.67 | | |  |
| **Panel D: Ages 60 plus** | | | | | | | | | | | | | | | | | |  |
| Age in years | | 67.63 | | 184 | | 67.48 | | 88 | | 67.77 | | | 96 | | | 0.75 | | |
| Male | | 0.38 | | 184 | | 0.39 | | 88 | | 0.36 | | | 96 | | | 0.77 | | |
| Recipient attended school | | 0.64 | | 184 | | 0.59 | | 88 | | 0.69 | | | 96 | | | 0.23 | | |
| Household has access to some toilet facilities | | 0.60 | | 184 | | 0.63 | | 88 | | 0.58 | | | 96 | | | 0.74 | | |
| Household has access to clean water source | | 0.24 | | 184 | | 0.26 | | 88 | | 0.23 | | | 96 | | | 0.77 | | |
| Household was affected by any shock | | 0.18 | | 184 | | 0.19 | | 88 | | 0.17 | | | 96 | | | 0.81 | | |
| Household size | | 6.55 | | 184 | | 6.63 | | 88 | | 6.49 | | | 96 | | | 0.80 | | |
| Total household monthly per capita expenditure (Zambian Kwacha) | | 36.31 | | 184 | | 39.15 | | 88 | | 33.71 | | | 96 | | | 0.12 | | |
| kaputa district | | 0.35 | | 184 | | 0.38 | | 88 | | 0.32 | | | 96 | | | 0.73 | | |
| shangombo district | | 0.27 | | 184 | | 0.27 | | 88 | | 0.26 | | | 96 | | | 0.92 | | |
| Illness | | 0.26 | | 184 | | 0.30 | | 88 | | 0.23 | | | 96 | | | 0.48 | | |
| Seek medical care | | 0.77 | | 47 | | 0.80 | | 25 | | 0.73 | | | 22 | | | 0.48 | | |
| Chronic illness | | 0.15 | | 183 | | 0.17 | | 87 | | 0.13 | | | 96 | | | 0.45 | | |
| Malaria | | 0.03 | | 184 | | 0.02 | | 88 | | 0.03 | | | 96 | | | 0.71 | | |
| Respiratory | | 0.06 | | 184 | | 0.08 | | 88 | | 0.04 | | | 96 | | | 0.26 | | |
| Diarrhoea | | 0.05 | | 184 | | 0.05 | | 88 | | 0.05 | | | 96 | | | 0.87 | | |
| Health expenditure (Zambian Kwacha) | | 8,846.19 | | 39 | | 2,095.24 | | 21 | | 16,722.29 | | | 18 | | | 0.28 | | |
| Self-assessed health | | 0.18 | | 182 | | 0.14 | | 86 | | 0.21 | | | 96 | | | 0.31 | | |

Notes: Bivariate regressions test difference between treatment and control groups. Self-assessed health only available for respondents 18 and older. Standard errors are clustered at the community level. Bold denotes significance at the alpha = 0.05 level.

**ZAMBIA MCT**

|  | | Pooled | | | | Control | | | | Treatment | | | | | |  | | |
| --- | --- | --- | --- | --- | --- | --- | --- | --- | --- | --- | --- | --- | --- | --- | --- | --- | --- | --- |
| Variables | | Mean | | N | | Mean | | N | | Mean | | | N | | | p-value | | |
| **Panel A: Children under 5** | | | | | | | | | | | | | | | | | | |
| Age in years | | 2.15 | | 1,662 | | 2.13 | | 798 | | 2.17 | | | 864 | | | 0.57 | | |
| Male | | 0.51 | | 1,662 | | 0.50 | | 798 | | 0.52 | | | 864 | | | 0.41 | | |
| Recipient attended school | | 0.70 | | 1,662 | | 0.72 | | 798 | | 0.69 | | | 864 | | | 0.38 | | |
| Household has access to some toilet facilities | | 0.94 | | 1,662 | | 0.92 | | 798 | | 0.96 | | | 864 | | | 0.08 | | |
| Household has access to clean water source | | 0.25 | | 1,662 | | 0.29 | | 798 | | 0.21 | | | 864 | | | 0.07 | | |
| Household was affected by any shock | | 0.60 | | 1,662 | | 0.62 | | 798 | | 0.58 | | | 864 | | | 0.54 | | |
| Household size | | 6.97 | | 1,662 | | 7.04 | | 798 | | 6.90 | | | 864 | | | 0.57 | | |
| Total household monthly per capita expenditure(Zambian Kwacha) | | 36.68 | | 1,662 | | 36.54 | | 798 | | 36.80 | | | 864 | | | 0.91 | | |
| Serenje district | | 0.61 | | 1,662 | | 0.60 | | 798 | | 0.61 | | | 864 | | | 0.92 | | |
| Illness | | 0.24 | | 1,662 | | 0.27 | | 798 | | 0.21 | | | 864 | | | 0.05 | | |
| Seek medical care | | 0.72 | | 389 | | 0.72 | | 214 | | 0.73 | | | 175 | | | 0.82 | | |
| Chronic illness | | 0.01 | | 1,651 | | 0.01 | | 797 | | 0.01 | | | 854 | | | 0.52 | | |
| Malaria | | 0.09 | | 1,662 | | 0.11 | | 798 | | 0.07 | | | 864 | | | 0.02 | | |
| Respiratory | | 0.06 | | 1,662 | | 0.07 | | 798 | | 0.06 | | | 864 | | | 0.54 | | |
| Diarrhoea | | 0.06 | | 1,662 | | 0.06 | | 798 | | 0.07 | | | 864 | | | 0.65 | | |
| Health expenditure (Zambian Kwacha) | | 346.87 | | 333 | | 337.79 | | 188 | | 358.64 | | | 145 | | | 0.91 | | |
| **Panel B: Ages 5-19** | | | | | | | | | | | | | | | | | | |
| Age in years | | 11.84 | | 7,334 | | 11.97 | | 3,614 | | 11.71 | | 3,720 | | | 0.06 | | |  |
| Male | | 0.53 | | 7,334 | | 0.54 | | 3,614 | | 0.53 | | 3,720 | | | 0.42 | | |  |
| Recipient attended school | | 0.68 | | 7,334 | | 0.69 | | 3,614 | | 0.66 | | 3,720 | | | 0.43 | | |  |
| Household has access to some toilet facilities | | 0.94 | | 7,334 | | 0.94 | | 3,614 | | 0.94 | | 3,720 | | | 0.85 | | |  |
| Household has access to clean water source | | 0.22 | | 7,334 | | 0.26 | | 3,614 | | 0.18 | | 3,720 | | | **0.02** | | |  |
| Household was affected by any shock | | 0.57 | | 7,334 | | 0.61 | | 3,614 | | 0.52 | | 3,720 | | | 0.12 | | |  |
| Household size | | 6.47 | | 7,334 | | 6.49 | | 3,614 | | 6.45 | | 3,720 | | | 0.86 | | |  |
| Total household monthly per capita expenditure(Zambian Kwacha) | | 39.68 | | 7,334 | | 40.85 | | 3,614 | | 38.54 | | 3,720 | | | 0.29 | | |  |
| Serenje district | | 0.55 | | 7,334 | | 0.57 | | 3,614 | | 0.53 | | 3,720 | | | 0.74 | | |  |
| Illness | | 0.10 | | 7,334 | | 0.10 | | 3,614 | | 0.09 | | 3,720 | | | 0.27 | | |  |
| Seek medical care | | 0.65 | | 673 | | 0.64 | | 355 | | 0.66 | | 318 | | | 0.82 | | |  |
| Chronic illness | | 0.01 | | 7,314 | | 0.01 | | 3,609 | | 0.01 | | 3,705 | | | 0.24 | | |  |
| Malaria | | 0.03 | | 7,334 | | 0.03 | | 3,614 | | 0.03 | | 3,720 | | | 0.79 | | |  |
| Respiratory | | 0.03 | | 7,334 | | 0.03 | | 3,614 | | 0.02 | | 3,720 | | | 0.47 | | |  |
| Diarrhoea | | 0.01 | | 7,334 | | 0.01 | | 3,614 | | 0.01 | | 3,720 | | | 0.55 | | |  |
| Health expenditure (Zambian Kwacha) | | 1,300.57 | | 554 | | 1,732.08 | | 309 | | 756.34 | | 245 | | | **0.02** | | |  |
| **Panel C: Ages 20-59** | | | | | | | | | | | | | | | | | |  |
| Age in years | | 35.92 | | 3,910 | | 35.81 | | 1,964 | | 36.03 | | | 1,946 | | | 0.68 | | |
| Male | | 0.35 | | 3,910 | | 0.35 | | 1,964 | | 0.34 | | | 1,946 | | | 0.82 | | |
| Recipient attended school | | 0.71 | | 3,910 | | 0.70 | | 1,964 | | 0.71 | | | 1,946 | | | 0.81 | | |
| Household has access to some toilet facilities | | 0.95 | | 3,910 | | 0.95 | | 1,964 | | 0.95 | | | 1,946 | | | 0.92 | | |
| Household has access to clean water source | | 0.24 | | 3,910 | | 0.27 | | 1,964 | | 0.20 | | | 1,946 | | | 0.07 | | |
| Household was affected by any shock | | 0.57 | | 3,910 | | 0.62 | | 1,964 | | 0.52 | | | 1,946 | | | 0.10 | | |
| Household size | | 6.28 | | 3,910 | | 6.32 | | 1,964 | | 6.24 | | | 1,946 | | | 0.74 | | |
| Total household monthly per capita expenditure(Zambian Kwacha) | | 44.30 | | 3,910 | | 43.98 | | 1,964 | | 44.62 | | | 1,946 | | | 0.81 | | |
| Serenje district | | 0.59 | | 3,910 | | 0.59 | | 1,964 | | 0.59 | | | 1,946 | | | 0.99 | | |
| Illness | | 0.14 | | 3,910 | | 0.13 | | 1,964 | | 0.14 | | | 1,946 | | | 0.61 | | |
| Seek medical care | | 0.64 | | 517 | | 0.61 | | 254 | | 0.66 | | | 263 | | | 0.38 | | |
| Chronic illness | | 0.04 | | 3,900 | | 0.05 | | 1,961 | | 0.04 | | | 1,939 | | | 0.40 | | |
| Malaria | | 0.02 | | 3,910 | | 0.02 | | 1,964 | | 0.03 | | | 1,946 | | | 0.26 | | |
| Respiratory | | 0.04 | | 3,910 | | 0.04 | | 1,964 | | 0.04 | | | 1,946 | | | 0.63 | | |
| Diarrhoea | | 0.01 | | 3,910 | | 0.01 | | 1,964 | | 0.01 | | | 1,946 | | | 0.77 | | |
| Health expenditure (Zambian Kwacha) | | 3,641.79 | | 404 | | 1,913.28 | | 196 | | 5,270.57 | | | 208 | | | 0.22 | | |
| Self-assessed health | | 0.52 | | 3,863 | | 0.53 | | 1,942 | | 0.52 | | | 1,921 | | | 0.69 | | |
| **Panel D: Ages 60 plus** | | | | | | | | | | | | | | | | | | |
| Age in years | | 71.70 | | 2,053 | | 71.94 | | 1,035 | | 71.45 | | | 1,018 | | | 0.27 | | |
| Male | | 0.33 | | 2,053 | | 0.34 | | 1,035 | | 0.33 | | | 1,018 | | | 0.61 | | |
| Recipient attended school | | 0.56 | | 2,053 | | 0.56 | | 1,035 | | 0.55 | | | 1,018 | | | 0.72 | | |
| Household has access to some toilet facilities | | 0.93 | | 2,053 | | 0.94 | | 1,035 | | 0.93 | | | 1,018 | | | 0.73 | | |
| Household has access to clean water source | | 0.23 | | 2,053 | | 0.27 | | 1,035 | | 0.18 | | | 1,018 | | | **0.01** | | |
| Household was affected by any shock | | 0.56 | | 2,053 | | 0.60 | | 1,035 | | 0.52 | | | 1,018 | | | 0.13 | | |
| Household size | | 4.59 | | 2,053 | | 4.66 | | 1,035 | | 4.51 | | | 1,018 | | | 0.54 | | |
| Total household monthly per capita expenditure(Zambian Kwacha) | | 56.04 | | 2,053 | | 56.47 | | 1,035 | | 55.60 | | | 1,018 | | | 0.84 | | |
| Serenje district | | 0.47 | | 2,053 | | 0.52 | | 1,035 | | 0.43 | | | 1,018 | | | 0.39 | | |
| Illness | | 0.30 | | 2,053 | | 0.32 | | 1,035 | | 0.27 | | | 1,018 | | | 0.12 | | |
| Seek medical care | | 0.59 | | 601 | | 0.61 | | 326 | | 0.57 | | | 275 | | | 0.47 | | |
| Chronic illness | | 0.12 | | 2,049 | | 0.13 | | 1,032 | | 0.11 | | | 1,017 | | | 0.56 | | |
| Malaria | | 0.04 | | 2,053 | | 0.04 | | 1,035 | | 0.03 | | | 1,018 | | | 0.28 | | |
| Respiratory | | 0.09 | | 2,053 | | 0.10 | | 1,035 | | 0.07 | | | 1,018 | | | 0.06 | | |
| Diarrhoea | | 0.03 | | 2,053 | | 0.02 | | 1,035 | | 0.03 | | | 1,018 | | | 0.59 | | |
| Health expenditure (Zambian Kwacha) | | 6,176.45 | | 426 | | 9,616.88 | | 243 | | 1,608.01 | | | 183 | | | 0.21 | | |
| Self-assessed health | | 0.16 | | 2,043 | | 0.17 | | 1,031 | | 0.14 | | | 1,012 | | | 0.16 | | |

Notes: Bivariate regressions test difference between treatment and control groups. Self-assessed health only available for respondents 18 and older. Standard errors are clustered at the community level. Bold denotes significance at the alpha = 0.05 level.

**ZIMBABWE**

|  | | Pooled | | | | Control | | | | Treatment | | | |  | |
| --- | --- | --- | --- | --- | --- | --- | --- | --- | --- | --- | --- | --- | --- | --- | --- |
| Variables | | Mean | | N | | Mean | | N | | Mean | | N | | p-value | |
| **Panel A: children under 5** | | | | | | | | | | | | | | | |
| Age | | 2.09 | | 1,748 | | 2.08 | | 588 | | 2.09 | | 1,160 | | 0.90 | |
| Household size | | 7.16 | | 1,748 | | 7.22 | | 588 | | 7.14 | | 1,160 | | 0.79 | |
| Main respondent female | | 0.71 | | 1,748 | | 0.67 | | 588 | | 0.73 | | 1,160 | | 0.09 | |
| Age of main respondent | | 45.75 | | 1,748 | | 47.85 | | 588 | | 44.82 | | 1,160 | | 0.07 | |
| Main respondent widowed | | 0.24 | | 1,748 | | 0.27 | | 588 | | 0.23 | | 1,160 | | 0.09 | |
| Main respondent divorced/separated | | 0.11 | | 1,748 | | 0.10 | | 588 | | 0.12 | | 1,160 | | 0.46 | |
| Main respondent ever attended school | | 0.74 | | 1,748 | | 0.72 | | 588 | | 0.75 | | 1,160 | | 0.33 | |
| Main respondent highest grade | | 4.78 | | 1,748 | | 4.54 | | 588 | | 4.89 | | 1,160 | | 0.20 | |
| Preventive care | | 0.63 | | 1,714 | | 0.64 | | 577 | | 0.63 | | 1,137 | | 0.82 | |
| Diarrhoea | | 0.18 | | 1,715 | | 0.20 | | 580 | | 0.18 | | 1,135 | | 0.47 | |
| Diarrhoea care | | 0.60 | | 308 | | 0.58 | | 112 | | 0.60 | | 196 | | 0.72 | |
| Fever | | 0.25 | | 1,715 | | 0.27 | | 580 | | 0.24 | | 1,135 | | 0.43 | |
| Fever care | | 0.53 | | 583 | | 0.54 | | 211 | | 0.53 | | 372 | | 0.80 | |
| Cough | | 0.37 | | 1,715 | | 0.41 | | 580 | | 0.35 | | 1,135 | | 0.06 | |
| Cough care | | 0.42 | | 733 | | 0.41 | | 262 | | 0.43 | | 471 | | 0.78 | |
| Health expenditure (USD) | | 0.82 | | 427 | | 0.66 | | 143 | | 0.91 | | 284 | | 0.50 | |
| **Panel B: Ages 5-19** | | | | | | | | | | | | | | | |
| Age | | 11.33 | | 7,020 | | 11.29 | | 2,331 | | 11.35 | | 4,689 | | 0.77 | |
| Household size | | 6.89 | | 7,020 | | 6.88 | | 2,331 | | 6.89 | | 4,689 | | 0.98 | |
| Main respondent female | | 0.67 | | 7,020 | | 0.66 | | 2,331 | | 0.68 | | 4,689 | | 0.43 | |
| Age of main respondent | | 51.26 | | 7,020 | | 52.16 | | 2,331 | | 50.87 | | 4,689 | | 0.29 | |
| Main respondent widowed | | 0.30 | | 7,020 | | 0.30 | | 2,331 | | 0.30 | | 4,689 | | 0.99 | |
| Main respondent divorced/separated | | 0.09 | | 7,020 | | 0.07 | | 2,331 | | 0.10 | | 4,689 | | 0.19 | |
| Main respondent ever attended school | | 0.65 | | 7,020 | | 0.66 | | 2,331 | | 0.65 | | 4,689 | | 0.76 | |
| Main respondent highest grade | | 3.98 | | 7,020 | | 3.88 | | 2,331 | | 4.03 | | 4,689 | | 0.48 | |
| Illness | | 0.15 | | 7,020 | | 0.15 | | 2,331 | | 0.16 | | 4,689 | | 0.63 | |
| Seek medical care | | 0.71 | | 1,112 | | 0.69 | | 356 | | 0.72 | | 756 | | 0.39 | |
| Chronic illness | | 0.04 | | 7,016 | | 0.03 | | 2,329 | | 0.04 | | 4,687 | | 0.17 | |
| Malaria | | 0.04 | | 7,020 | | 0.04 | | 2,331 | | 0.05 | | 4,689 | | 0.46 | |
| Respiratory | | 0.04 | | 7,020 | | 0.04 | | 2,331 | | 0.03 | | 4,689 | | 0.89 | |
| Diarrhoea | | 0.01 | | 7,020 | | 0.02 | | 2,331 | | 0.01 | | 4,689 | | 0.38 | |
| Illness | | 0.15 | | 7,020 | | 0.15 | | 2,331 | | 0.16 | | 4,689 | | 0.63 | |
| Seek medical care | | 0.71 | | 1,112 | | 0.69 | | 356 | | 0.72 | | 756 | | 0.39 | |
| Chronic illness | | 0.04 | | 7,016 | | 0.03 | | 2,329 | | 0.04 | | 4,687 | | 0.17 | |
| Self-assessed health | | 0.12 | | 7,007 | | 0.11 | | 2,325 | | 0.12 | | 4,682 | | 0.60 | |
| Malaria | | 0.04 | | 7,020 | | 0.04 | | 2,331 | | 0.05 | | 4,689 | | 0.46 | |
| Respiratory | | 0.04 | | 7,020 | | 0.04 | | 2,331 | | 0.03 | | 4,689 | | 0.89 | |
| Diarrhoea | | 0.01 | | 7,020 | | 0.02 | | 2,331 | | 0.01 | | 4,689 | | 0.38 | |
| Health expenditure (USD) | | 1.90 | | 1,112 | | 1.95 | | 356 | | 1.88 | | 756 | | 0.92 | |
| Self assessed health | | 0.12 | | 7,007 | | 0.11 | | 2,325 | | 0.12 | | 4,682 | | 0.60 | |
| **Panel C: Ages 20-59** | | | | | | | | | | | | | | | |
| Age | | 36.22 | | 3,060 | | 36.72 | | 1,050 | | 35.99 | | 2,010 | | 0.07 | |
| Household size | | 6.89 | | 3,060 | | 6.90 | | 1,050 | | 6.89 | | 2,010 | | 0.97 | |
| Main respondent female | | 0.63 | | 3,060 | | 0.63 | | 1,050 | | 0.63 | | 2,010 | | 0.94 | |
| Age of main respondent | | 47.20 | | 3,060 | | 48.38 | | 1,050 | | 46.64 | | 2,010 | | 0.13 | |
| Main respondent widowed | | 0.22 | | 3,060 | | 0.24 | | 1,050 | | 0.21 | | 2,010 | | 0.18 | |
| Main respondent divorced/separated | | 0.08 | | 3,060 | | 0.08 | | 1,050 | | 0.09 | | 2,010 | | 0.57 | |
| Main respondent ever attended school | | 0.72 | | 3,060 | | 0.71 | | 1,050 | | 0.72 | | 2,010 | | 0.76 | |
| Main respondent highest grade | | 4.62 | | 3,060 | | 4.46 | | 1,050 | | 4.70 | | 2,010 | | 0.33 | |
| Illness | | 0.28 | | 3,060 | | 0.24 | | 1,050 | | 0.29 | | 2,010 | | **0.04** | |
| Seek medical care | | 0.74 | | 794 | | 0.72 | | 260 | | 0.75 | | 534 | | 0.50 | |
| Chronic illness | | 0.15 | | 3,059 | | 0.14 | | 1,050 | | 0.15 | | 2,009 | | 0.47 | |
| Malaria | | 0.04 | | 3,060 | | 0.04 | | 1,050 | | 0.04 | | 2,010 | | 0.78 | |
| Respiratory | | 0.07 | | 3,060 | | 0.05 | | 1,050 | | 0.08 | | 2,010 | | 0.02 | |
| Diarrhoea | | 0.02 | | 3,060 | | 0.02 | | 1,050 | | 0.02 | | 2,010 | | 0.71 | |
| Illness | | 0.28 | | 3,060 | | 0.24 | | 1,050 | | 0.29 | | 2,010 | | **0.04** | |
| Seek medical care | | 0.74 | | 794 | | 0.72 | | 260 | | 0.75 | | 534 | | 0.50 | |
| Chronic illness | | 0.15 | | 3,059 | | 0.14 | | 1,050 | | 0.15 | | 2,009 | | 0.47 | |
| Self assessed health | | 0.26 | | 3,058 | | 0.24 | | 1,048 | | 0.27 | | 2,010 | | 0.11 | |
| Malaria | | 0.04 | | 3,060 | | 0.04 | | 1,050 | | 0.04 | | 2,010 | | 0.78 | |
| Respiratory | | 0.07 | | 3,060 | | 0.05 | | 1,050 | | 0.08 | | 2,010 | | **0.02** | |
| Diarrhoea | | 0.02 | | 3,060 | | 0.02 | | 1,050 | | 0.02 | | 2,010 | | 0.71 | |
| Health expenditure (USD) | | 7.21 | | 794 | | 6.32 | | 260 | | 7.55 | | 534 | | 0.63 | |
| Self-assessed health | | 0.26 | | 3,058 | | 0.24 | | 1,048 | | 0.27 | | 2,010 | | 0.11 | |
| **Panel D: Ages 60 plus** | | | | | | | | | | | | | | | |
| Age | | 72.09 | | 2,668 | | 72.00 | | 946 | | 72.13 | | 1,722 | | 0.86 | |
| Household size | | 4.65 | | 2,668 | | 4.72 | | 946 | | 4.62 | | 1,722 | | 0.74 | |
| Main respondent female | | 0.61 | | 2,668 | | 0.60 | | 946 | | 0.61 | | 1,722 | | 0.67 | |
| Age of main respondent | | 67.12 | | 2,668 | | 66.96 | | 946 | | 67.20 | | 1,722 | | 0.84 | |
| Main respondent widowed | | 0.38 | | 2,668 | | 0.38 | | 946 | | 0.38 | | 1,722 | | 0.91 | |
| Main respondent divorced/separated | | 0.06 | | 2,668 | | 0.04 | | 946 | | 0.07 | | 1,722 | | **0.02** | |
| Main respondent ever attended school | | 0.46 | | 2,668 | | 0.53 | | 946 | | 0.43 | | 1,722 | | **0.03** | |
| Main respondent highest grade | | 2.16 | | 2,668 | | 2.51 | | 946 | | 2.00 | | 1,722 | | **0.01** | |
| Illness | | 0.51 | | 2,668 | | 0.50 | | 946 | | 0.52 | | 1,722 | | 0.56 | |
| Seek medical care | | 0.70 | | 1,385 | | 0.69 | | 477 | | 0.70 | | 908 | | 0.78 | |
| Chronic illness | | 0.26 | | 2,668 | | 0.26 | | 946 | | 0.25 | | 1,722 | | 0.78 | |
| Malaria | | 0.05 | | 2,668 | | 0.03 | | 946 | | 0.05 | | 1,722 | | **0.02** | |
| Respiratory | | 0.10 | | 2,668 | | 0.08 | | 946 | | 0.11 | | 1,722 | | 0.10 | |
| Diarrhoea | | 0.04 | | 2,668 | | 0.05 | | 946 | | 0.03 | | 1,722 | | 0.21 | |
| Illness | | 0.51 | | 2,668 | | 0.50 | | 946 | | 0.52 | | 1,722 | | 0.56 | |
| Seek medical care | | 0.70 | | 1,385 | | 0.69 | | 477 | | 0.70 | | 908 | | 0.78 | |
| Chronic illness | | 0.26 | | 2,668 | | 0.26 | | 946 | | 0.25 | | 1,722 | | 0.78 | |
| Self-assessed health | | 0.62 | | 2,666 | | 0.59 | | 944 | | 0.63 | | 1,722 | | 0.19 | |
| Malaria | | 0.05 | | 2,668 | | 0.03 | | 946 | | 0.05 | | 1,722 | | **0.02** | |
| Respiratory | | 0.10 | | 2,668 | | 0.08 | | 946 | | 0.11 | | 1,722 | | 0.10 | |
| Diarrhoea | | 0.04 | | 2,668 | | 0.05 | | 946 | | 0.03 | | 1,722 | | 0.21 | |
| Health expenditure (USD) | | 4.86 | | 1,385 | | 5.70 | | 477 | | 4.51 | | 908 | | 0.50 | |
| Self-assessed health | | 0.62 | | 2,666 | | 0.59 | | 944 | | 0.63 | | 1,722 | | 0.19 | |

Notes: Bivariate regressions test difference between treatment and control groups. Self-assessed health only available for respondents 18 and older. Standard errors are clustered at the community level. Bold denotes significance at the alpha = 0.05 level.
